# Reactivity of Petrobactin and Its Sulfonated Derivatives with Iron and Their Determination by Isotopic Saturation Fast Size-Exclusion Chromatography–Inductively Coupled Plasma Mass Spectrometry (ICP-MS)

**DOI:** 10.1021/acsomega.5c06088

**Published:** 2025-08-27

**Authors:** Katarzyna Kińska, Isaura Caceres, Abdel Khouk, Sophie Nolivos, Régis Grimaud, Laurent Ouerdane, Joanna Szpunar, Ryszard Łobinski

**Affiliations:** † 49605 University of Warsaw, Faculty of Chemistry, Pasteura 1, 02-093 Warsaw, Poland; ‡ Institute of Analytical Sciences and Physico-Chemistry for Environment and Materials (IPREM-UMR5254) UPPA/CNRS, Hélioparc, 2, Av. Pr. Angot, Pau 64053, France; § Warsaw Institute of Technology, Faculty of Chemistry, Chair of Analytical Chemistry, Noakowskiego 3, 00-664 Warsaw, Poland

## Abstract

Petrobactin is a bis-catechol siderophore, synthesized by *Marinobacter nauticus* (formerly *Marinobacter hydrocarbonoclasticus*), an important oil-degrading bacterium that proliferates in oil-polluted marine ecosystems. The complexes formed by petrobactin and its sulfonated derivatives with iron were, for the first time, chromatographically separated and identified by mass spectrometry. Conditions for the separation of the iron complexes using reversed-phase HPLC and size-exclusion LC were optimized. A method for quantifying petrobactin and its sulfonated derivatives has been developed. The analytical procedure is based on the saturation of the apo form of the siderophore with isotopically enriched iron, followed by its separation by ultraperformance size-exclusion chromatography with ICP-MS detection. The method is characterized by a detection limit of 0.03 ± 0.01 and 0.02 ± 0.01 μmol L^–1^, for petrobactin and sulfonated derivatives, respectively. Conditions of the formation of iron complexes were discussed in terms of iron source and pH. The complexation reaction was the fastest when iron was supplied as citrate or malate and when it occurred at pH 8. The monosulfonated derivative bound iron significantly faster than petrobactin itself, unlike the disulfonated derivative.

## Graphical abstract

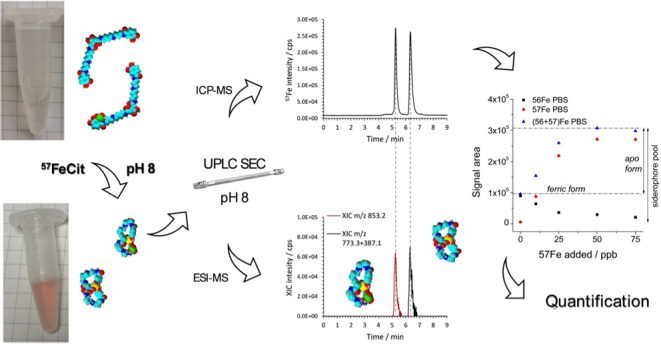

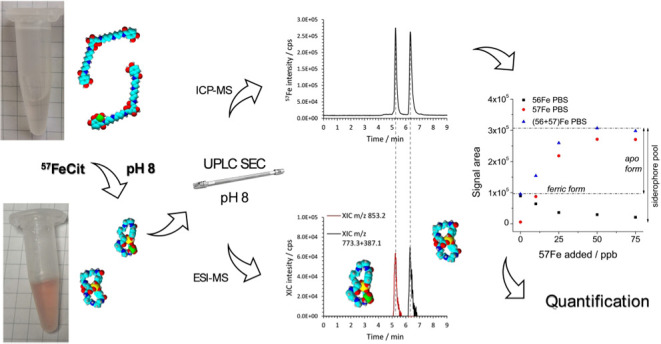

## 1 Introduction

Because of its rich coordination chemistry and the coordination-relevant Fe^3+^/Fe^2+^ redox potential relationship, iron is a prosthetic element of many enzymes that plays a vital role in bacteria and other organisms.^1–4^ The acquisition of iron by bacteria is, therefore, fundamental for their growth and activity.

In aerobic conditions, in neutral and alkaline environments, iron is present mainly as the thermodynamically stable Fe^3+^ which forms insoluble ferric (oxyhydr)­oxides, limiting its availability to microorganisms.^5,6^ Therefore, bacteria developed a system for the iron solubilization based on the synthesis and secretion of siderophores, high-affinity ligands for Fe^3+^, followed by the import of Fe^3+^–siderophore complexes into the cell.^7,8^ To date, more than 500 siderophores have been isolated and their structure determined.^9,10^

Petrobactin was first isolated and identified from the marine bacterium *M. nauticus* SP17 *(*formerly *M. hydrocarbonoclasticus* SP17*)* cultures.^11,12^ *M. nauticus* SP17 exhibits the ability to use as a carbon and energy source, hardly water-soluble compounds, such as long-chain alkanes, triglycerides, fatty acids, and wax esters.^13^ Members of the genus *Marinobacter* play an important role in the bioremediation of marine ecosystems, degrading various hydrocarbon compounds present in crude oil.^14^ Petrobactin is a mixed catechol-hydroxy-carboxylate siderophore^12,15^ prone to sulfonation. The catechol moiety in petrobactin is the 3,4-dihydroxybenzoate (3,4-DHB), whereas the vast majority of known catechol siderophores use the 2,3-DHB isomer (Figure 1).^15–17^ The 3,4-DHB configuration, observed uniquely in petrobactin and its sulfonated derivatives,^18,19^ is atypical in the siderophore world, and can play a role in the physiology of petrobactin-producing bacteria.^20,21^ For instance, petrobactin (but not its sulfonated derivatives) is produced not only by *Marinobacter* strains but also by terrestrial pathogenic bacteria of the species *Bacillus anthracis* and *Bacillus cereus*.^22–24^ Because of the presence of the 3,4-DHB isomer, petrobactin is not recognized by the host protein siderocalin and thus evades the immune system, being considered to be a stealth siderophore.^25^

**Figure 1:**
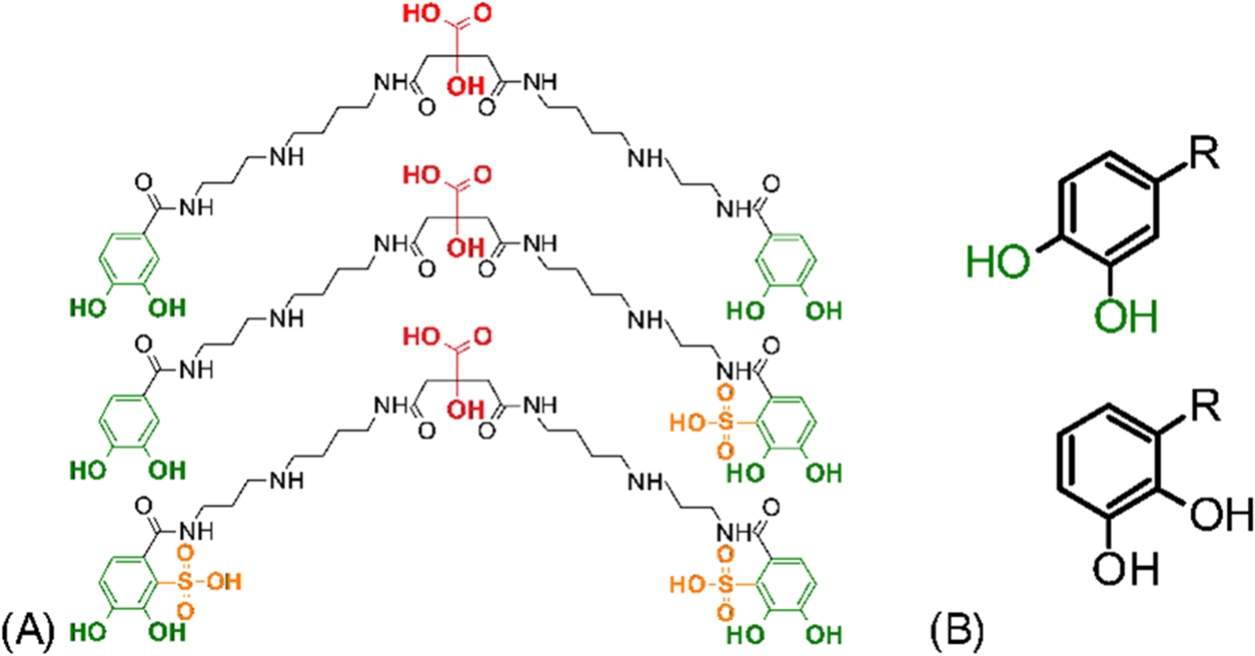
Structural formulas of petrobactin and monosulfonated and disulfonated derivatives (A). 3,4-DHB and 2,3-DHB isomers (B). Different colors were used to highlight key parts of the molecule: orange to mark the sulfonation site, green to show differences in the catechol moiety (specific to petrobactin 3,4-DHB), and red for the carboxyl moiety.

Sulfonation of the aromatic ring, which is a structural modification specific to marine siderophores,^23^ can affect the hydrophilicity of the siderophore, increasing its solubility in water and altering the stability constant of the iron complexes, by stabilizing the catechol ring against oxidation.^19^

Petrobactin and its two sulfonated derivatives have been extensively characterized by nuclear magnetic resonance (NMR) spectroscopy and various types of mass spectrometry (MS).^12,16,18,19,26^ The reports, however, were limited to the apo siderophore forms. To our best knowledge, no molecular evidence of the existence of [PB-2H+Me­(III)]^+^ (a charge-reduced ion) of PB complex, observed for Ga^3+^,^26^ exists for Fe^3+^. Attempts to determine the [PB-2H+Fe]^+^ form alongside the doubly charged form by MS have been unsuccessful, indicating its absence or (photo)­instability.^12,26^ Data on sulfonated derivatives are even more scarce, and no MS evidence of their existence has yet been demonstrated.

The goal of this research was to provide the first molecular evidence of the formation of iron complexes by sulfonated derivatives of petrobactin and confront it with limited data on the complexation of petrobactin itself. For this purpose, the reactivity of PB and its derivatives toward iron was extensively studied in different conditions in terms of the source of iron and chemical conditions. Particular attention was paid to the pH of complexation and the source of iron to promote rapid and effective binding. The saturation of petrobactin and its sulfonated derivatives by isotopically enriched iron was optimized to become the basis of the development of a method for the quantitative determination of the ferric and apo forms of siderophores produced by *M. nauticus* SP17 by size-exclusion chromatography ICP-MS.

## 2 Materials and Methods

### 2.1 Reagents, Standards, and Solutions

Deionized water from a Milli-Q Type 1 system (Millipore, Belford, MA) was used throughout. Acetonitrile (ACN, ≥99.9%, LC-MS grade), ammonium acetate (AmAc, ≥98% for molecular biology), methanol (MeOH, ≥98%, LC-MS grade), ammonium formate (≥99%, for mass spectrometry), and formic acid (98–100%, LiChropur for LC-MS) were provided by Sigma-Aldrich (www.sigmaaldrich.com). Quantification of apo and complexed forms of siderophores was carried out using a standard solution of isotopically enriched ^57^Fe (CRM, 100 ± 4 mg L^–1^; 2.85% ^56^Fe, 95.34% ^57^Fe, 1.78% ^58^Fe, ISC SCIENCE, www.isc-science.com). For double labeling, alongside ^57^Fe, isotopically enriched ^58^Fe (99.81% enrichment; STB Isotope Germany GMBH, www.stb-isotope.com) was used, after its previous dissolution in aqua regia to a concentration of 50 mg mL^–1^. Citric acid, malic acid, and ethylenediaminetetraacetic acid (EDTA) were used as ligands to prepare the spiking solutions of iron complexes at known iron concentrations. The spiking solutions were prepared in ammonium acetate using ammonia to raise the pH.

### 2.2 Siderophore Samples

Petrobactin and its derivatives were obtained from *M. nauticus* *SP17* cultured and subsequently purified according to the procedure described in detail in the Supporting Information. In brief, after 5 days of cultivation, siderophores in the culture were sorbed on Amberlite XAD-2 resin and subsequently eluted with methanol. The fractions tested positive by CAS assay^27^ were dried, redissolved in water, and purified by RP-LC. The siderophore’s purity was analyzed by qNMR using the ERETIC2 (Electronic Reference To access In vivo Concentrations) approach (Wider and Dreier,^28^), as described in detail in SI.

### 2.3 Instrumentation

Chromatographic separations were carried out using an Agilent 1200 (www.agilent.com) or a Dionex Ultimate 3000 RS (www.thermofisher.com) LC system. The exit of the column was connected to ICP-MS (Agilent 7700X ICP-MS, www.agilent.com) or ESI-MS (Thermo Scientific Orbitrap Fusion Lumos Tribrid, www.thermofisher.com) analyzers for qualitative and quantitative studies. Petrobactin and its derivatives were separated on SEC (Acquity UPLC protein BEH SEC column, 125Å, 1.7 μm, 4.6 × 150 mm) and RP (Acquity UPLC BEH C18 column, 1.7 μm, 2.1 × 150 mm) columns from Waters (www.waters.com).

### 2.4 Procedures

#### 2.4.1 Formation of Complexes with Isotopically Enriched Iron

Petrobactin, sulfonated petrobactin, or disulfonated petrobactin solutions (20–100 μL, 1–10 μmol L^–1^ based on NMR measurements) were diluted with AmAc solution (20–40 μL, 100 mmol L^–1^, pH 8.8–9.5) to maintain a slightly alkaline reaction environment (pH ∼ 8). Subsequently, an ^57^Fe-enriched solution was added, without prior ion complexation or in the form of citrate/malate complexes (pH 4–5) or EDTA (pH ∼ 8), and the solution was diluted with water to 200 μL. Solutions were prepared in advance to ensure iron binding before chromatographic separation and analysis. As both the complex with EDTA and petrobactin (and its derivatives) are photounstable, the incubations were conducted under light-restricted conditions using light-tight containers.^6,12,29^ The donor complexes were prepared by mixing a stock solution of citrate/malate/EDTA with an ^57^Fe stock solution in the presence of AmAc.

The kinetics of the formation of complexes of petrobactin and its derivatives was studied following the addition of an excess (0.06 μg) of isotopically enriched ^57^Fe standard (as citrate) to 50 μL (10 μmol L^–1^) of a siderophore solution in AmAc (pH 8–9.5), with a final volume adjusted to 200 μL. Samples were separated immediately after mixing and after progressively longer incubation to monitor complex formation over time.

#### 2.4.2 Separation of Iron Complexes

The complexes were separated isocratically on an SEC column with 10 mmol L^–1^ AmAc (pH 7.9–8.0) as the mobile phase (0.3 mL min^–1^) at 25 °C within 15 min using ICP-MS or ESI-MS detection. The injection volume was 5–10 μL. For Fast-SEC–ESI-MS, 10 mM AmAc pH 8.9 in 90% ACN or MeOH (0.1–0.3 mL min^–1^) was added postcolumn to improve ionization, using a three-way connector between the column exit and the electrospray source. Separations on the RP column, adapted from a previous study,^30^ were carried out in gradient elution mode at 0.3 mL min^–1^, either in acidic (A: 5 mmol L^–1^ ammonium formate in 0.1% formic acid; B: ACN-MeOH 90–10% in 0.1% formic acid; 0–2 min 5% B, 2–3 min up to 98% B, 3–6 min 98% B, 6–7 min down to 5% B; 40 °C) or basic conditions (A: 10 mmol L^–1^ AmAc pH 8; B: 10 mmol L^–1^ AmAc pH 8 in 90% ACN; 0–2 min 3% B, 2–3 min up to 100% B, 3–6 min 100% B, 6–7 min down to 3% B; 30 °C) with ESI-MS detection only.

#### 2.4.3 ICP-MS Conditions

All measurements were performed using nickel sampler and skimmer cones, with an RF power of 1550 W, carrier gas flow of 1.05 L min^–1^, and makeup gas flow of 0.1 L min^–1^ (optimized if necessary). Other experimental conditions, torch position, ion lenses, and cell parameters were adjusted daily according to a standard optimization protocol. Hydrogen (4–4.5 mL min^–1^) was used as a reaction gas to suppress spectral interference (from polyatomic molecular ions). The isotopes ^56^Fe, ^57^Fe, and ^58^Fe were monitored throughout the study.

#### 2.4.4 ESI-MS Conditions

Determinations were carried out at a resolution of 240,000 over a range of *m*/*z* 200/250–1200 with an ionization energy of 3500 V (positive ionization mode). The ESI-MS operating parameters were optimized to avoid hydrolysis of complexes and doubly charged ions: shielding/sheath gas (50 Arb, arbitrary units), Aux gas (10 Arb), sweep gas (1 Arb), ion transfer tube temperature (300–350 °C), vaporizer temperature (300–350 °C), RF lens (20–100%), maximum injection time (100 ms). MS/MS analysis was performed for apo forms and Fe-enriched complexes using higher-energy C-trap dissociation (HCD) at collision energies of 20–45.

#### 2.4.5 Iron-Siderophores Identification

Sample enrichment with two heavier iron isotopes, ^57^Fe and ^58^Fe, modified the natural isotopic pattern of the element and allowed a targeted survey utilizing the pattern scoring parameter in Compound Discoverer 3.3 (Thermo Scientific). Fragmentation data were used to confirm the presence of each siderophore and its complex.

#### 2.4.6 Quantification

Ferric–siderophore complexes were quantified by SEC-ICP-MS. The concentrations of the siderophore–iron complexes and apo forms were determined from saturation curves obtained for petrobactin and its derivatives (20–100 μL, 1–10 μmol L^–1^). The siderophore-containing solution (20–40 μL of AmAc, 100 mmol L^–1^, pH 8.0–9.5) was spiked with 2–60 μL of 1.0 ppm of isotopically enriched iron (^57^Fe citrate of EDTA complex) and made up with water to 200 μL. Samples were separated at least 8 h after mixing to ensure Fe–siderophores formation.

## 3 Results and Discussion

### 3.1 Formation of the Fe–PB Complex: Preliminary Experiments

#### 3.1.1 Infusion and Reversed-Phase HPLC–ESI-MS

The initial experiments aimed at probing the detection of the complexes of petrobactin and its derivatives by reversed-phase ESI-MS in literature conditions: formic acid/acetonitrile-methanol gradient elution.^31–33^ The purified petrobactin (PB) and its monosulfonated derivative (PBS) were spiked with a mixture of ^57^Fe/^58^Fe, as reported elsewhere.^34^ The use of the pair of ^57^Fe/^58^Fe isotopes facilitates the search for the complexes due to the formation of a modified iron isotopic pattern.^34,35^ However, regardless of the amount of iron added (50–100 ng mL^–1^), only apo forms were found (Figure S1). Therefore, to promote complex formation and its stability throughout the chromatographic separation, ammonium acetate of pH 8, similar to the *M. nauticus* *SP17* culture growing media, was used in this study, as pH seems to be critical for the formation and the stability of the iron–PB complex. It was shown by Zhang et al. that gradual acidification of a solution containing the completely soluble Fe­(III)­PB^3–^ anion (pH 11.3), was shown to cause absorbance changes linked to the appearance of partially protonated Fe­(III)­PBH^2–^ and Fe­(III)­PBH_2_^–^ forms until the neutral form (violet-blue color) precipitated at pH 6.2.^21^ The generation of Fe–PB complexes was carried out in water–methanol solution or AmAc (pH 6.8) by mixing FeCl_3_ (in 0.1 mol L^–1^ HCl) and PB in a 1:1 ratio.^26,36^ Elsewhere, the complex was prepared in the dark from a DMSO stock solution of the free ligand and FeCl_3_ in phosphate buffer (pH 7.4).^37^

The mass spectra (Figure 2), acquired after reversed-phase separation at pH 8, indicate the binding of iron in a complex with petrobactin and sulfonated petrobactin. Both the apo form and the Fe­(III)-PB ions are, to a great extent, double-charged. Besides single- and double-charged ions of apo (*m*/*z* 719.4 and 360.2) and ^57^Fe/^58^Fe petrobactin (Table 1, *m*/*z* 773.3/774.3 and 387.1/387.6), the ions of the decarboxylated complex with a mass difference of 44/22 (*m*/*z* 729.3/730.3 and 365.1/365.6) were observed with the same retention time (in-source decarboxylation) (Figure 2a,b). In-source decarboxylation is an example of in-source fragmentation, when, among others, pseudomolecular ions [M + H]^+^ lose the CO_2_ group, and in general is an inherent phenomenon in ESI-MS. Note that the two signals (773.3 and 729.3) contained the ^57^Fe/^58^Fe characteristic isotopic pattern created for this experiment (Figure 2d). The ionization was different for the sulfonated derivative and its complexes, which were nearly exclusively present as monocharged ions; hence, in this case, only the [M + H]^+^ ions were investigated (Figure 2c). Again, in addition to the pseudomolecular ion of the apo form (*m*/*z* 799.3) and ^57^Fe/^58^Fe-complexed sulfonated petrobactin (*m*/*z* 853.2/854.2), a decarboxylated form (*m*/*z* 809.2/810.2) characterized by the same isotopic pattern was observed. The ion of *m*/*z* 673.3, resulting from the decarboxylation and oxidation of unbound petrobactin, mentioned by Barbeau,^12^ appeared at a different retention time than PB and its ^57^Fe complex, but its XIC intensity was more than 2 orders of magnitude lower than PB. When separating at pH 2, the two masses were recorded at almost the same retention time, and the one after decarboxylation and oxidation was 3 orders of magnitude smaller than the main ion. Its equivalent for the sulfonated form (*m*/*z* = 753.3) was recorded only during separation at pH 2. The XIC intensity was 2 orders of magnitude lower than the parent ion. Thus, despite the possible difference in ionization of the different forms of siderophores, we can conclude that, with appropriate conditions of complex preparation, their degradation under ultraviolet (UV) light does not pose a serious problem in that kind of study. Our results are consistent with those reported in the literature. Fourier transform ion cyclotron resonance (FTICR) mass spectrometry (MS) and sustained off-resonance irradiation collision-activated dissociation tandem mass spectrometry (SORI-CAD MS/MS) used for petrobactin characterization revealed the production of ions (*m*/*z* 719.36 and 360.18) corresponding to [M + H]^+^ and [M + 2H]^2+^, respectively.^38^ The group compared SORI-CAD and ECD fragmentations of metal-complexed petrobactin generated by ESI from a solution containing water and methanol, without any chromatographic separation. They pointed out that the fragmentation spectra of the complex formed with Fe­(III), in contrast to Ga­(III), do not contain a charge-reduced precursor ion (*m*/*z* 772), which they explain by the easy loss of CO_2_/COOH from the ferric molecule. The authors refer to studies by Barbeau et al., who demonstrated UV-induced instability of the complex, leading to its decarboxylation and oxidation and the formation of a molecule with a mass difference of 46 (*m*/*z* 673).^11,12,26^

**Figure 2:**
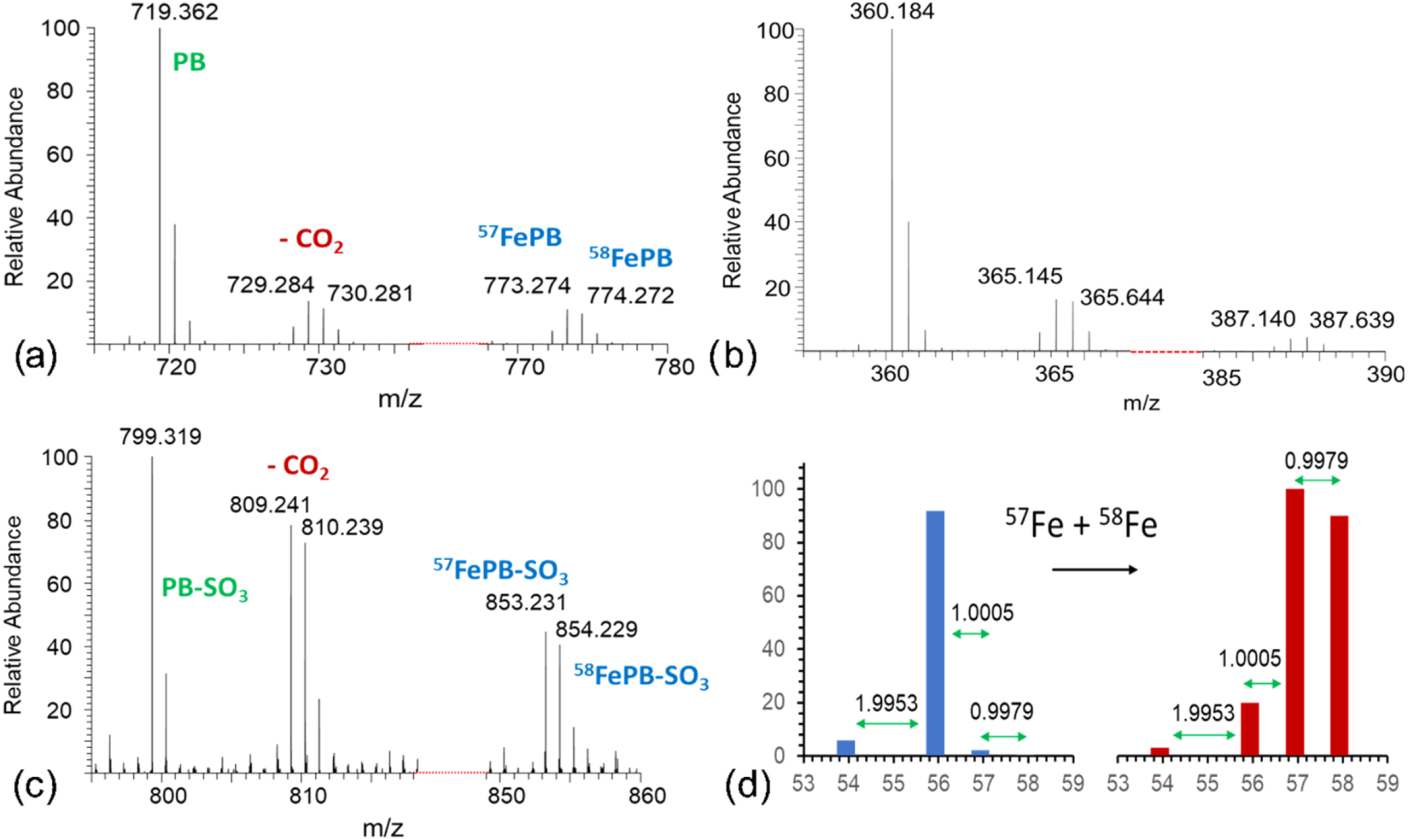
Mass spectra of petrobactin and sulfonated petrobactin after their complexation with ^57^Fe/^58^Fe: (a) petrobactin [M + H]^+^, *m*/*z* 719.362 complexed with isotopically enriched Fe, *m*/*z* 773.274/774.272 and after a loss of the CO_2_ group (in-source decarboxylation, *m*/*z* 729.284/730.281); (b) petrobactin [M+2H]^2+^, *m*/*z* 360.184 complexed with isotopically enriched Fe, *m*/*z* 387.140/387.639 and after a loss of the CO_2_ group (*m*/*z* 365.145/365.644); (c) sulfonated petrobactin [M + H]^+^, *m*/*z* 799.319 complexed with isotopically enriched Fe, *m*/*z* 853.231/854.229 and after a loss of the CO_2_ group (809.241/810.239); (d) isotopic pattern of natural (blue) and ^57^Fe/^58^Fe-enriched (red) iron.

**Table 1:** Comparison of m/z of Pseudomolecular Ions of Petrobactin (PB) and Sulfonated Petrobactin (PBS) Obtained by RP-ESI-MS at pH 8

*m*/*z*	petrobactin	sulfonated petrobactin
[M + H]^+^/[M+2H]^2+^	719.4/360.2	799.3
[M-2H+^57^Fe]^+^/[...]^2+^	773.3/387.1	853.2
[M-2H+^58^Fe]^+^/[...]^2+^	774.3/387.6	854.2
[M-2H+^57^Fe-CO_2_]^+^/[...]^2+^	729.3/365.1	809.2
[M-2H+^58^Fe-CO_2_]^+^/[...]^2+^	730.3/365.6	810.2
[M+H–CO_2_–H_2_]^+^	673.3	753.3^a^

a Obtained after separation at pH 2.

To sum up, reverse-phase chromatographic separation with a mobile phase of pH 8 allowed the detection of both the apo- and the complexed forms of petrobactin and its sulfonated derivative (Figure 3). However, at this pH, both siderophores and their complexes were very poorly retained on the RP column. A similar effect was observed when attempting to retain the complex on an SPE column, using a hydrophilic–lipophilic balanced reversed-phase sorbent (HLB). When the FePB and FePBS complexes prepared at pH 8 were introduced onto the SPE column, significantly more of the compounds were found in the solution passing through the column (90 and 85%, respectively), and only a fraction after elution with the MeOH solution (10 and 15%). A test carried out for apo forms at pH 2 and pH 8 showed that in alkaline media, the sulfonated form passes through the column to a lesser extent than petrobactin (65 and 82%, respectively). At acidic pH, both compounds sorb quantitatively and are eluted by more than 99% in the next step (MeOH/0.3% FA). The use of SPE therefore requires the sample to be acidified before it is introduced onto the column. Hence, purification/preconcentration of petrobactin and its derivatives on SPE, proposed elsewhere for the desalting,^39^ is limited to apo forms, as the acidification step necessary for the quantitative binding of compounds prevents observation of Fe-bound forms. Hence, it was necessary to find an alternative chromatographic method, allowing both the separation of individual siderophores and their separation from the matrix (matrix simplification).

**Figure 3:**
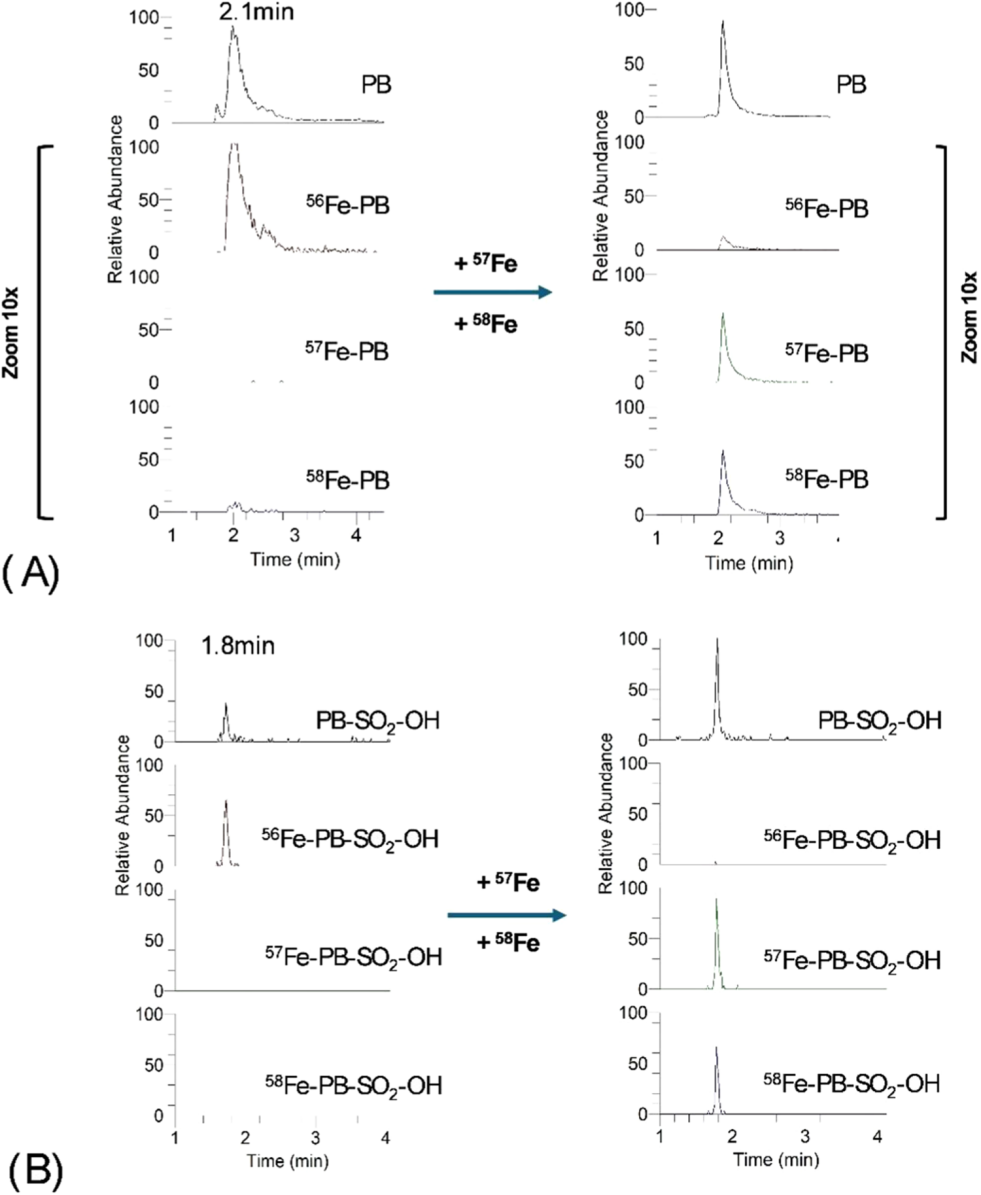
Reversed-phase ESI-MS chromatograms of petrobactin (a) and its sulfonated form (b) before and after spiking with ^57^Fe/^58^ Fe-enriched standard. Separations in gradient elution on the RP column at a flow rate of 0.3 mL min-1 (A: 10 mmol L^–1^ ammonium acetate pH 8; B: 10 mmol L^–1^ ammonium acetate pH 8 in 90% ACN; 0–2 min 3% B, 2–3 min up to 100% B, 3–6 min 100% B, 6–7 min down to 3% B; 30 °C).

#### 3.1.2 Fast Size-Exclusion Chromatography ESI-MS

Size-exclusion chromatography, traditionally used for separation based on molecular size, also involves a number of poorly understood secondary interactions with the stationary phase, allowing the separation of small, similarly sized metal complexes, as demonstrated elsewhere for the separation of metal–siderophore complexes produced by soil bacteria^34^ and in hyperaccumulating plants.^40^ Indeed, we found it possible to baseline-separate petrobactin and its monosulfonated derivative using fast-SEC (Figure 4). This allowed the use of ICP-MS detection, which offers efficient and stable ionization of iron, regardless of its complex. At the same time, the chromatography reduced the salt load and prevented interference due to the buildup of salt crystals at the cone interface and the consequent reduction in sensitivity, reported elsewhere.^39^ However, as separation of mono- and disulfonated derivatives was not entirely possible in optimized conditions, the use of ESI-MS was necessary to verify if only one sulfonated form was present in the samples (Figure S2). Therefore, the detection conditions were optimized. To ensure efficient ionization of petrobactin and sulfonated petrobactin complexes without their decomposition at the source, the following conditions were proposed after optimization (described in detail in the Supporting Information): postcolumn acetonitrile flow of 100 μL min^–1^, RF 30, ITT 300 °C, VP 325 °C, ITTT 300 °C.

**Figure 4:**
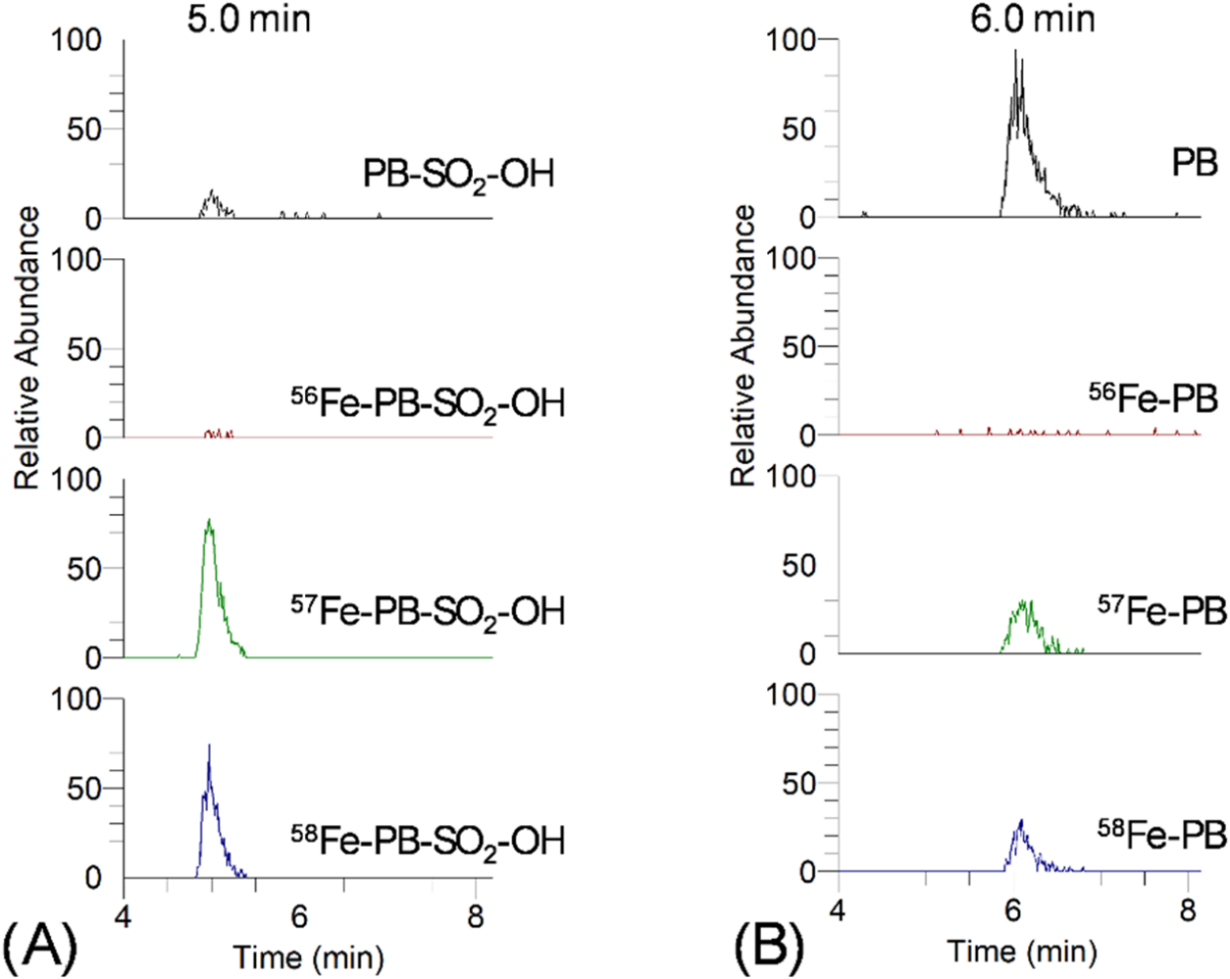
Size-exclusion ESI-MS XIC chromatograms of petrobactin (a) and sulfonated petrobactin (b), after spiking with ^57^Fe/^58^ Fe-enriched standards. Isocratic separations were performed on the SEC column with 10 mmol L^–1^ ammonium acetate (pH 7.9–8.0) at a flow rate of 0.3 mL min^–1^, 25 °C.

Unlike the sulfonated forms, petrobactin consistently showed a signal from the apo form, even in the presence of excess iron. This observation may indicate complex dissociation in the ion source, with the extent of dissociation being strongly dependent on the measurement conditions applied. In contrast, data for disulfonated petrobactin complexation suggest that under the conditions used in SEC measurements, only the ferric complex was present. Signals at *m*/*z* 933.187 and 934.185, corresponding to the ^57^Fe and ^58^Fe complexes, respectively, increased with rising iron concentration (the siderophore concentration remained constant during sample preparation). Meanwhile, in a solution prepared in the same way but analyzed on an RP column under acidic conditions, the apo form (*m*/*z* 879.275) is predominantly detected (Figure S5).

### 3.2 Effect of the Iron Source on the Acquisition of Iron by Petrobactin and Its Derivatives

#### 3.2.1 Choice of the Iron Source

To enable the quantification of PB and its derivatives, the iron complex formation must be both rapid and quantitative. However, in order to track the kinetics of the formation of individual complexes, iron binding should not be immediate. Furthermore, the iron source itself should remain stable and should not undergo hydrolysis to a significant extent. Various sources of iron were evaluated, including a diluted stock solution of isotopically enriched ^57^Fe in 2% HNO_3_ and its complexes with citrate, malate, and EDTA. The free ^57^Fe standard was successfully used for the quantitative determination of mixed citrate–malate complexes of iron in coconut water at pH 5.5,^35^ while citrates were applied in previous studies of siderophore complexation.^34,41^ The initial assay design assumed that all isotopically enriched iron would form complexes with an excess of ligand. This assumption held when the sample preparation and separation were conducted at a sufficiently low pH to maintain Fe in a soluble form. However, under the more alkaline conditions (pH 8), obtained after mixing with AmAc/NH_3,_ at which the above studies were conducted, iron remains soluble only when strongly complexed. Nevertheless, all of the studied forms of iron dissociate/hydrolyze to some degree. Size-exclusion ICP-MS proved well suited to study the transfer of ^57^Fe to PB and sulfonated PB (Figure 5).

**Figure 5:**
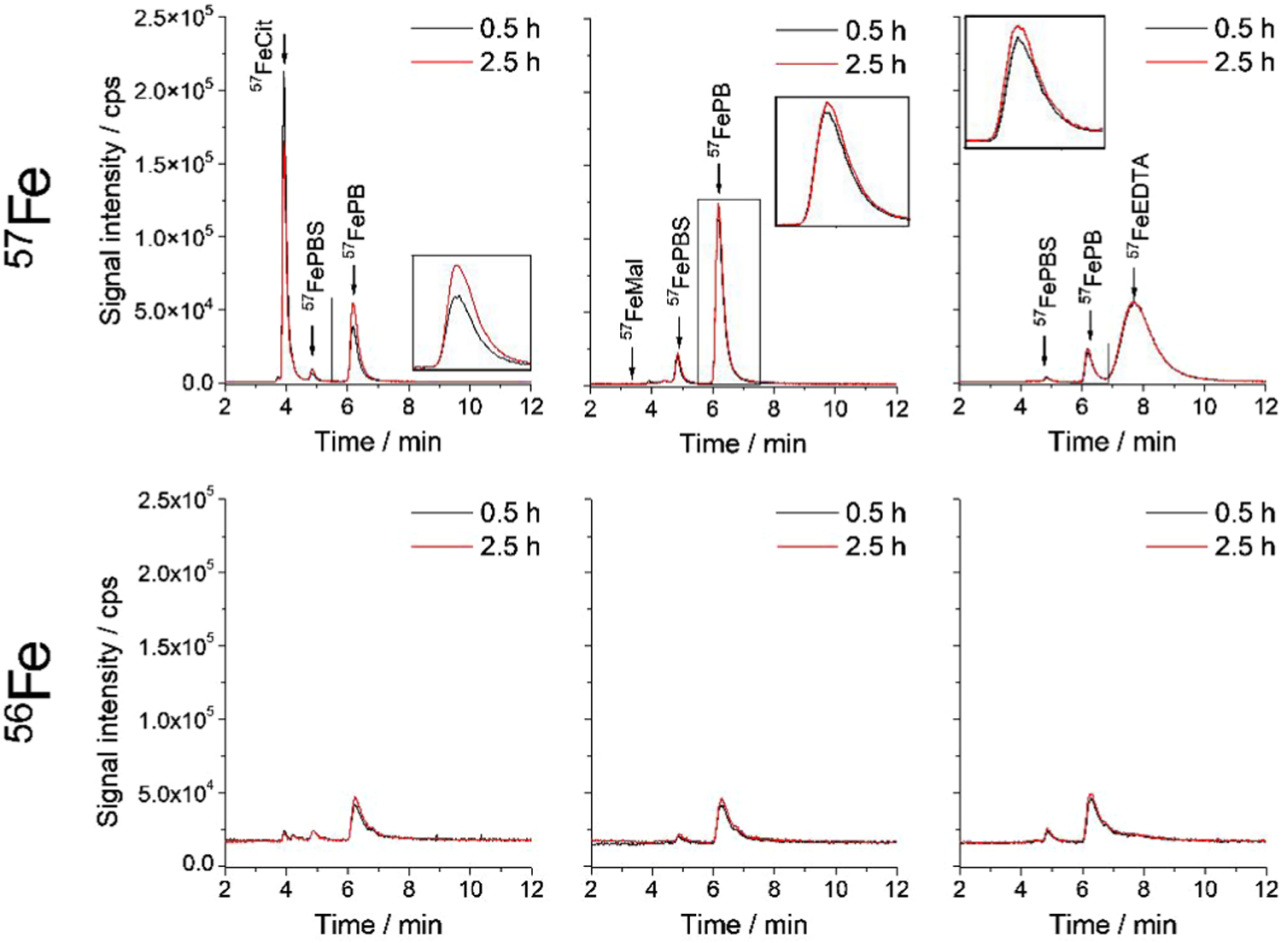
Size-exclusion ICP-MS chromatograms of the petrobactin-sulfonated petrobactin mixture (100 μL) incubated with different sources of iron–citrates (^57^FeCit), malates (^57^FeMal), and EDTA (^57^FeEDTA) (100 ng mL^–1^) over 0.5 and 2.5 h. The upper panel shows a chromatogram of compounds complexed with ^57^Fe, while the bottom panel shows compounds complexed with ^56^Fe.

The fastest complexation was observed when ^57^FeMal was used as the iron source. However, its application has two notable drawbacks. First, complete complexation occurred less than 30 min after mixing, which may not enable the differentiation of complexation kinetics between petrobactin and its sulfonated derivatives. Second, the malate complex was less stable under alkaline conditions, leading to greater iron loss during chromatography when added in excess, although the losses were still considerably lower than in the case of noncomplexed ^57^Fe form. In fact, after injection of ^57^FeMal onto the column, no signal was recorded, which would confirm that binding of Fe­(III) to the siderophore was much faster than its hydrolysis. In contrast, the stability of the ^57^FeEDTA resulted in an almost 5-fold increase in equilibration time of exchange, and even then, the complexation was not quantitative. The results confirm the higher availability of iron from ferric citrate compared with ferric EDTA, as it was shown elsewhere for algal cell growth.^42^

Of the systems tested, the citrate complex proved to be the optimal source of iron for our study as it reacts rapidly with PB and sulfonated PB while allowing the complexation kinetics to be monitored over a relatively short time (Figure 5). Between the first and second chromatographic separations spanning approximately 2 h, with the first chromatogram recorded about 30 min after complex preparation, the signal of ^57^FePB formed following the addition of ^57^FeCit significantly increased.

#### 3.2.2 Effect of the Complexation Conditions

After selection of ^57^FeCit as the optimal iron source, the influence of pH on siderophore complexation was studied in greater detail. The isotopically enriched ^57^Fe standard was mixed with a citrate solution at an initial pH of 3–4 and then combined with AmAc solution at pH values of approximately 5.5 and 8. The resulting ^57^FeCit solutions were added to a mixture of petrobactin and sulfonated petrobactin, together with 0.1 mol L^–1^ AmAc solution of pH 8.0, 8.7, or 9.5 and incubated for 1 h. Signals originating from ^57^FePB and ^57^FePBS of similar intensity were recorded under each condition, indicating that the complexation efficiency of the studied siderophores under the given pH values did not differ significantly, particularly for PB (301 ± 13 μM, RSD < 5%) (Figure S6). However, the preparation of ^57^FeCit at pH 8 resulted in better PB complexation during the test period (309 ± 9 μM), compared with lower ^57^FeCit pH (285–293 μM). Moreover, an effect of the pH on the stability of ^57^FeCit itself was observed when increasing the pH above 8. The iron standards were added to the mixture at equal concentrations, while the signal coming from the unreacted ^57^FeCit was recorded only at the lowest pH tested. Based on these results, pH 8 was considered optimal for the preparation of siderophore complexes, while also being better suited for injection onto the column.

The degree of complexation of PB and PBS by ^57^Fe from ^57^FeCit was checked over time. Chromatograms of siderophore–iron citrate mixture were recorded within 8 h of mixing the components (Figure 6). A significant increase in the intensity of the ^57^Fe–siderophore-derived signal and a decrease in the intensity of the ^57^FeCit-derived signal were observed (Figure 6). During the investigated time span, the signal areas of petrobactin (*RT* = 6.2 min, C) and sulfonated petrobactin (*RT* = 4.9 min, B) grow around 2.5 times, while the signal of iron citrate (*RT* = 3.9 min, A) decreases of about 3 times (Figure 6). At the same time, signals of ^56^Fe-complexed ligands were monitored (Figure 6, right panel). A decrease in the intensity of ^56^FePB, which could indicate a substitution of natural iron with the heavier isotope, was not noticed. A slight increase in the ^56^FePB signal may be the result of instrument drift over time or PB complexation with ^56^Fe present in the isotopically enriched ^57^Fe standard (2.85% of ^56^Fe).

**Figure 6:**
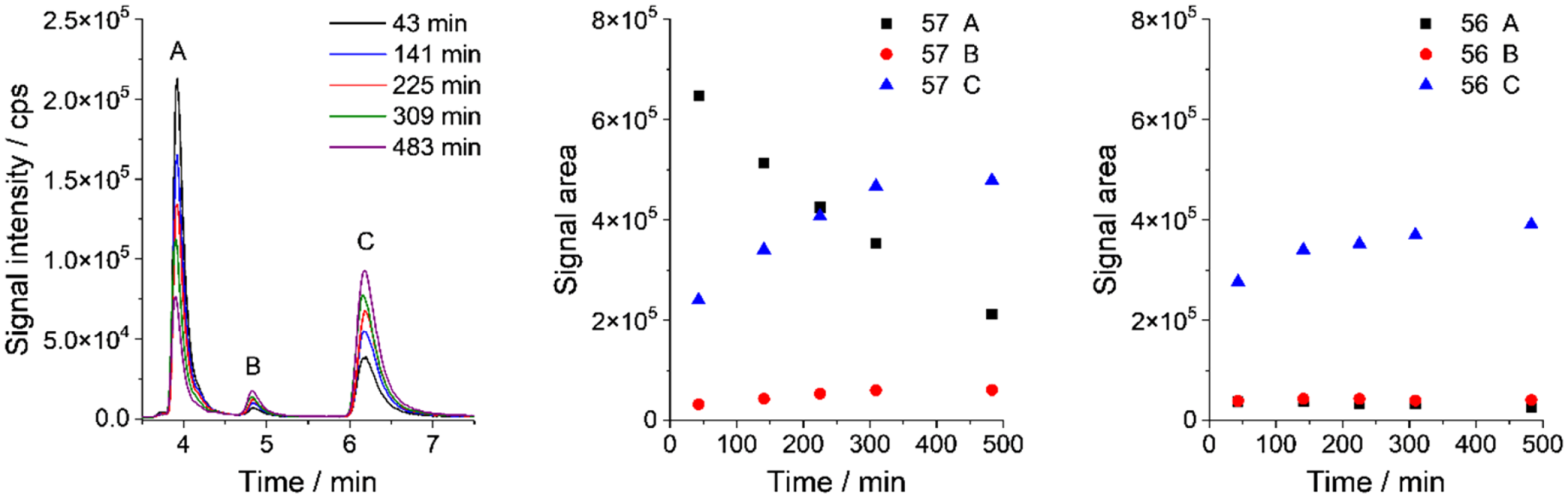
Petrobactin (PB)/sulfonated petrobactin (PBS) complexation as a function of incubation time: Size-exclusion ICP-MS chromatograms of PB/PBS mixture (100 μL) incubated with ^57^Fe citrates (50 ng mL^–1^), left panel; changes in the peak area of ^57^Fe-complexed compounds ^57^FeCit (A), ^57^FePBS (B), ^57^FePB (C) in time, middle panel; changes in the peak area of ^56^Fe-complexed compounds ^56^FeCit (A), ^56^FePBS (B), ^56^FePB (C) in time, right panel.

Subsequently, the effect of the ^57^FeCit quantity on PB complexation over time was studied (Figure S7). At first glance, even with a significant excess of ^57^FeCit (Figures S8–S10), no isotopic exchange of ^56^Fe to ^57^Fe in the original petrobactin complex was observed, but only the formation of new complexes with free siderophore. However, when we calculate and compare the ratios of ^57^FePB/^56^FePB and ^57^FePBS/^56^FePBS, we can see that they change over time, especially for the sulfonated form. When 100 ppb of ^57^Fe was added to the PB–PBS mixture (Figure S10), the ratios increased from 85 to 97% for petrobactin and from 122 to 160% for sulfonated petrobactin; hence, the possibility of isotope exchange should not be overlooked.

#### 3.2.3 Kinetics of Formation of Iron Complexes with Petrobactin, Sulfonated Petrobactin, and Disulfonated Petrobactin

The kinetics of ligand exchange can be influenced by many factors. We evaluated siderophore iron binding as a function of time after the addition of excess ^57^Fe (Figure 7). We examined differences in the binding time of Fe by disulfonated petrobactin, depending on the form of Fe used (Figure 8). And we studied siderophore iron binding as a function of ^57^Fe concentration, until full saturation was achieved (Figure 9). A comparison of the complexation of petrobactin and its two sulfonated derivatives under identical conditions, with an excess of the complexing reagent (^57^FeCit), shows that the complexes with monosulfonated petrobactin form most readily, reaching maximum signal intensity in less than 1 h after mixing (Figure 7). In the case of petrobactin, the required time was twice as long, while for disulfonated petrobactin, the signal plateau had not been reached even after 12 h, indicating that these siderophores bind iron more slowly. Complementary experiments conducted with the disulfonated siderophore showed that approximately 80% of complexation rate, compared with ^57^FeCit→PBS2, was obtained after 2 days of incubation, while more than 90% after 16 days when ^57^FeEDTA was used as an iron source (Figure 8). The same studies demonstrated that complexation rate does not exceed 60%, even after 16 days of incubation, when not complexed ^57^Fe was used as the iron source. To the best of our knowledge, there is no specific study in the literature linking the kinetics of petrobactin–Fe complex formation with biofilm iron capture or higher growth rates in polluted settings. However, faster complexation of sulfonated petrobactin with Fe would likely increase iron availability in microenvironments like biofilms. Since iron is a limiting nutrient, especially in oil-polluted marine areas, this could confer enhanced ecological fitness to *M. nauticus* by supporting better colonization, growth, and pollutant degradation.

**Figure 7:**
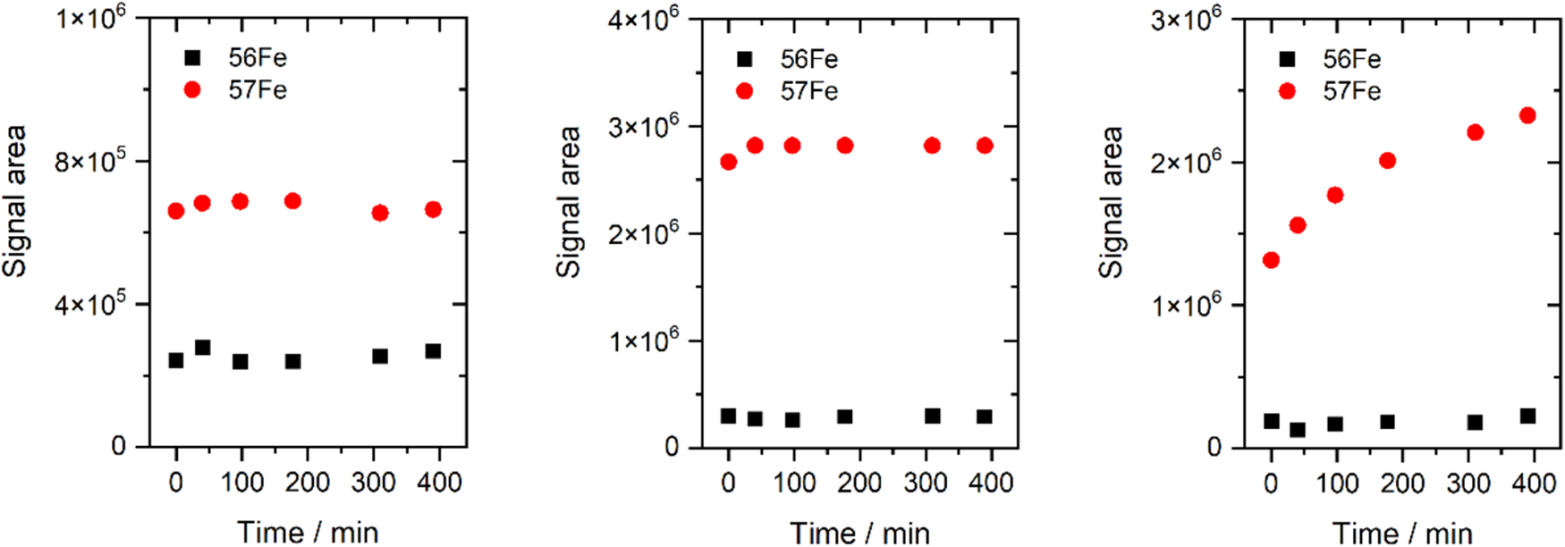
Comparison of the formation kinetics of ferric complexes of petrobactin (left panel), sulfonated petrobactin (middle panel), and disulfonated petrobactin (right panel) at high excess ^57^FeCit. Signal areas were normalized to the signal area for sulfonated petrobactin after 40 min of incubation to minimize instrument drift.

**Figure 8:**
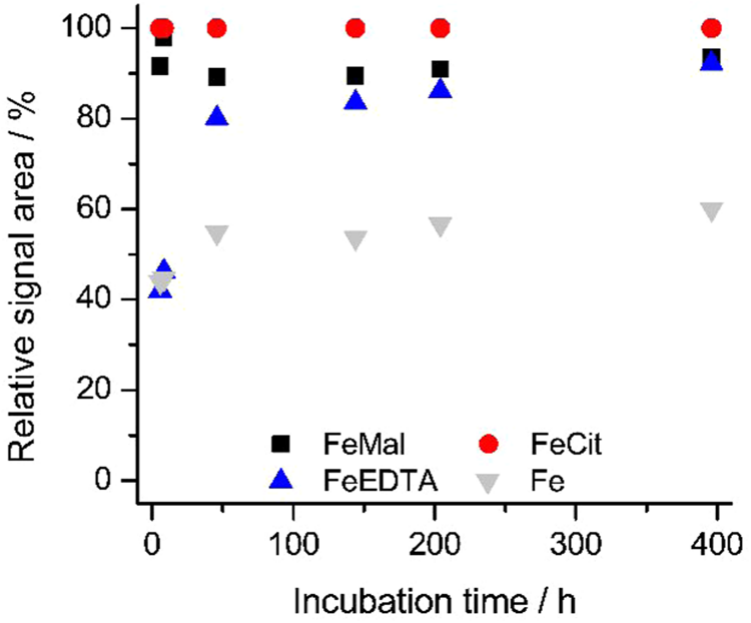
Disulfonated petrobactin complexation via incubation time (6–400 h) and ^57^Fe source.

**Figure 9:**
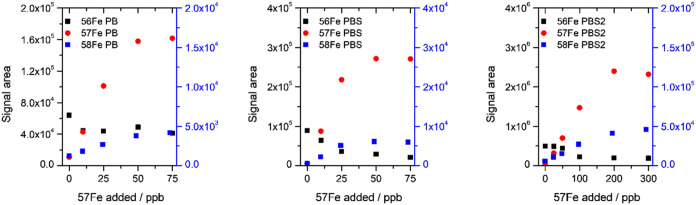
Comparison of petrobactin (PB), sulfonated petrobactin (PBS), and disulfonated petrobactin (PBS2) complexation with ^57^FeCit. Signal areas of ^56^Fe and ^57^Fe complexes are shown on the left axis and ^58^Fe on the right axis.

At a constant concentration of ^57^FeCit added to the siderophore solution, the signals originated from natural iron complex (^56^FePB–^56^FePBS–^56^FePBS2) remained relatively stable over time. This indicates either the absence of isotopic exchange with the heavier isotope or that such an exchange occurs rapidly and is completed immediately following the spike. To further investigate the possibility of isotopic exchange across all studied siderophores, the signal areas corresponding to complexes with ^56^Fe, ^57^Fe, and ^58^Fe were compared following successive additions of the ^57^FeCit complex. Notably, the complex contained not only ^57^Fe but also 2.85% ^56^Fe and 1.78% ^58^Fe in petrobactin, sulfonated petrobactin, or disulfonated petrobactin (Figure 9). The data showed that signals from the ^56^Fe complexes were higher before spiking and decreased upon successive additions of ^57^FeCit. Comparison between unspiked and spiked samples revealed that depending on the siderophore concentration, different amounts of ^57^FeCit were required to reach a plateau. However, even with a large excess of the heavier isotope, the signal from the ^56^Fe form never dropped to the background level (i.e., 0). The drop of ^56^Fe–siderophore signal was observed after the addition of 10 ng mL^–1^ of ^57^FeCit into PB solution, while further additions did not have any significant effect on the system. In the PBS system, the highest drop was observed after 25 ng mL^–1^, but final areas/concentration of the ^57^Fe complex were also about twice higher. In the PBS2 system, the most significant ^56^Fe signal drop was noticed between 50 and 100 ng mL^–1^ of the introduced ^57^FeCit. It appears that ^56^FePB undergoes the least isotopic exchange, with enriched ^57^Fe/^58^Fe complexes forming in the presence of excess ligands or siderophore. In the end, approximately 20% of the siderophore remains bound to ^56^Fe, as the final signal retains about 65% of the initial intensity. For the sulfonated forms, isotopic exchange appears to occur to a greater extent. Only about 7% of the complexes remain bound to the lighter isotope, with final signals corresponding to about 24 and 38% of the initial ones, for mono- and disulfonated petrobactin, respectively. Together with the previous results, we conclude that isotope exchange did not occur within this group of siderophores. However, it does not appear to be time-dependent, as suggested by the time-resolved studies. Instead, the exchange likely takes place immediately after the initial addition of the labeled iron to the sample. Nevertheless, time-dependent data showing an increase in the ^56^Fe–petrobactin signal may suggest that the system gradually approached an equilibrium, as both ^56^Fe and ^57^Fe signals increase over time. To conclude, although the siderophores form very strong complexes, in the case of petrobactin with the formation constant of 10^43^, isotopic exchange with heavier isotopes may still occur, likely due to their tendency to form stronger, less dissociable bonds.^21,43^

### 3.3 Quantitative Determination of Petrobactin and Its Forms by Isotopic Saturation and Exchange

The ultimate goal of this work is to propose a method for quantifying petrobactin derivatives, particularly in ferric form, which has not been possible until now. The SEC-ICP-MS method studied, after saturation of siderophores with isotopically enriched iron, is a good candidate for this purpose (high repeatability of recorded chromatograms within several hours of analysis, with RSD of 3.2 and 2.5% for petrobactin iron complexes and its sulfonated derivative, respectively, *n* = 4). A cross-method comparison with NMR and RP-ESI-MS, which are techniques often used in quantitative determinations of siderophores, will allow for the reliability of the obtained results to be verified.

To determine the concentration of siderophores using the SEC-ICP-MS method, a series of siderophore solutions was prepared in advance, at least 48 h prior to analysis, with varying additions of isotopically enriched iron until the free siderophores were fully saturated (Figures 9 and[Fig fig10]). In the initial range, the signal increase for ^57^Fe and ^58^Fe isotopes was linear (with the signals for ^58^Fe 2 orders of magnitude lower than for ^57^Fe due to the small contribution of ^58^Fe to the isotopically enriched standard, 1.78%). Above a certain concentration, a plateau of the signal area for ferric-siderophores was observed (with the simultaneous appearance of a signal from the unreacted ^57^FeCit, Figure 10), indicating consumption of the whole pool of apo siderophores. The signal area for the ^56^Fe isotope corresponded to the original content of the complex, while the sum of the signal areas from ^56^Fe and ^57^Fe isotopes at the saturation point indicates total siderophore concentration (^58^Fe signals were negligible if not added separately). The difference between the sum of the signal areas from ^56^Fe and ^57^Fe isotopes before spiking and at the saturation point makes it possible to determine the concentration of the apo form of the studied siderophores (Figure 11).

**Figure 10:**
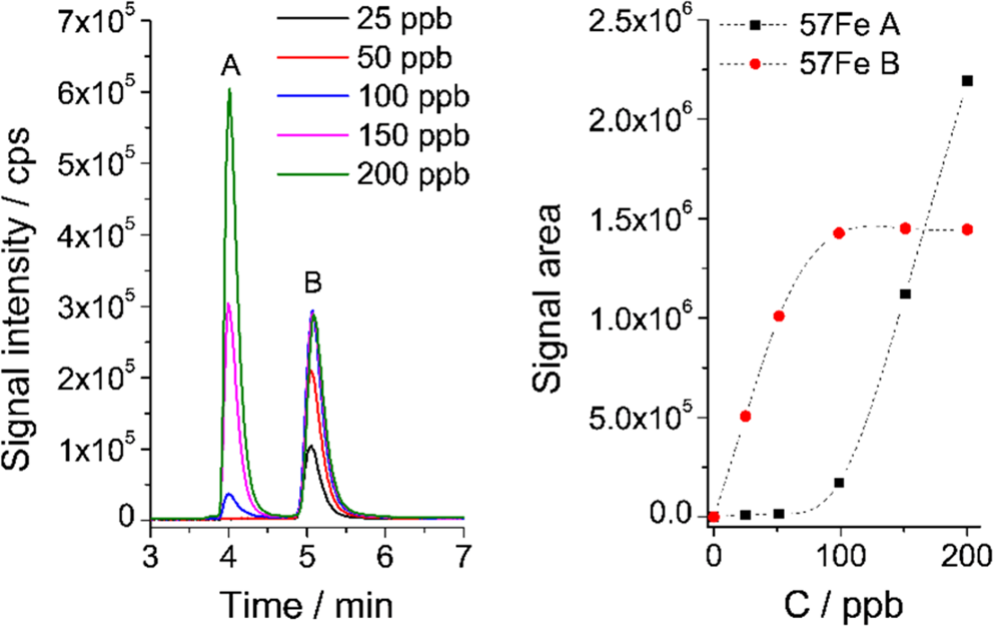
Sulfonated petrobactin complexation as a function of the ^57^FeCit concentration: Size-exclusion ICP-MS chromatograms of the sulfonated petrobactin (B) incubated with isotopically enriched iron source ^57^FeCit (A, 25–200 ng mL^–1^), left panel; saturation curves of sulfonated petrobactin after addition of ^57^FeCit, right panel.

**Figure 11:**
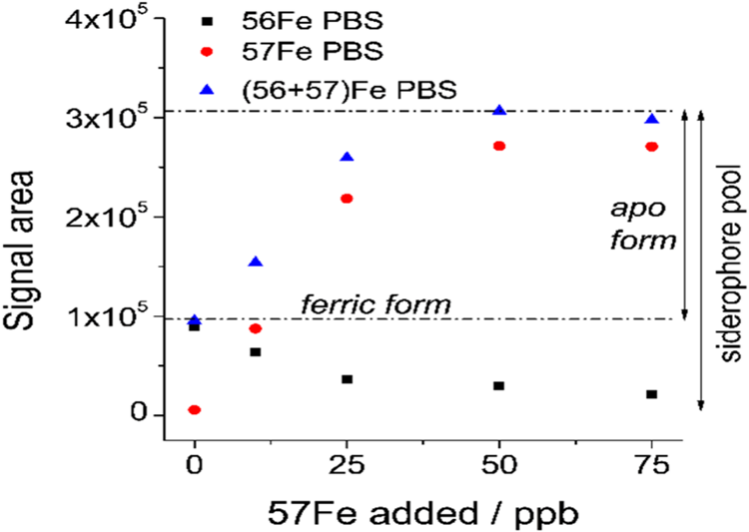
Scheme of calculation method of siderophore concentration calculation, together with ferric and apo forms of siderophore amount, on the example of monosulfonated petrobactin (PBS) complexation.

To determine the total amount of iron complexed by petrobactin and its sulfonated derivatives, two approaches can be used. The first one requires preparation of the saturation curve described above. In the initial stage, all iron added into the sample participates in the complex formation, though it is stabilized in the solution. The signal area of one of the recorded signals is then assigned to the concentration of ^57^Fe introduced into the solution. This value is subsequently used as a reference to calculate the concentration of iron in all compounds of the analyzed samples. The second approach is based on the addition of ^57^Fe in excess, derived from preliminary data. In such a case, only the part of iron that participates in siderophore complexation is well stabilized. The other part of ^57^Fe from the complex with citrates (or malates) can hydrolyze and precipitate, so the summaric area of all signals in the recorded chromatogram cannot be assigned to the added amount of ^57^Fe. In that case, ^57^FeEDTA solution can be applied to serve as a reference point for iron concentration, as in the alkaline conditions, it is the most stable form of iron (except siderophores). The known concentration of the ^57^FeEDTA complex can be assigned to its surface area, and the concentrations of ^56^Fe and ^57^Fe in each sample may be determined from the ratio of the signal surface areas of the enriched siderophore samples, as it was described for the first approach. Whichever approach is chosen, the next step is to calculate the total molar concentration of each siderophore. The values indicating the Fe content in the formed complexes should be subsequently divided by the molar mass of the respective isotope, leading to a molar concentration of the iron, which is equivalent to the molar concentration of the siderophore. By adding up the obtained values, information on the siderophore concentration in the analyzed sample can be retrieved. Taking into account all dilutions to which the sample was subjected during preparation, it is possible to calculate the total molar siderophores concentration in the original fraction.

The detection limit of the applied SEC-ICP-MS method was determined using the formula LOD = A̅_0_ + 3.3SD­(A_0_), where A̅_0_ is the mean area of noise measured within the retention time of a given peak, and SD­(A_0_) is the standard deviation of that value. Limits of detection for each siderophore were determined for data collected from 3 measurement days. The average LOD values were 0.02 ± 0.01 and 0.03 ± 0.01 μmol L^–1^, for PBS and PB, respectively (per molar concentration of siderophore). Owing to spectral interference, the signals recorded for ^56^Fe (0.7 ± 0.3 and 1.3 ± 0.5 ng mL^–1^ for PBS and PB, respectively) contributed more to this value than ^57^Fe (0.3 ± 0.2 and 0.4 ± 0.3 ng mL^–1^ for PBS and PB, respectively).

Data obtained from SEC-ICP-MS measurements for petrobactin and monosulfonated petrobactin (Table 2) indicate that the sulfonated form is present at a concentration that is more than 2.5 times higher than that of petrobactin itself. There was a difference between the PB values measured by NMR (646 μmol L^–1^) and SEC-ICP-MS (364 μmol L^–1^), which was not observed for sulfonated PB. A possible explanation is the greater instability of PB compared with its sulfonated form during the few months that elapsed between the NMR and SEC-ICP-MS measurements. The data were also compared with those obtained by ESI-MS after separation on a reversed-phase column in standard, acidic pH, typically used in the determinations of apo siderophores. With the initial assumption that the two forms exhibit similar ionization after separation at acidic pH, similar results were obtained as in SEC-ICP-MS. ESI-MS calculations were based on the calibration curve of the sulfonated form, XIC area change for *m*/*z* 799 vs PBS concentration (determined by SEC-ICP-MS). The concentration for PB was then determined from the slope based on XIC of the sum of *m*/*z* 360 and 719 ([M+2H]^2+^ and [M + H]^+^ ions).

**Table 2:** Comparison of Petrobactin and Sulfonated Petrobactin Concentration (μmol L^–1^) in Purified Fractions from *M. nauticus* Cultures from ICP-MS and ESI-MS Measurements

	NMR quantified fractions			fractions
	NMR	SEC-ICP-MS	RP-ESI-MS	SEC-ICP-MS
petrobactin	646	364 ± 24 (*n* = 5)	348 ± 26 (*n* = 23)^a^	363 ± 18 (*n* = 6)
sulfonated petrobactin	935	958 ± 22 (*n* = 11)	976 ± 79 (*n* = 13)^a^	533 ± 2 (*n* = 13)
disulfonated petrobactin	-	-	-	291 (*n* = 1)

a Quantification based on the concentration of PBS determined by SEC-ICP-MS (958 μmol L^–^).

## 4 Conclusions

This study characterized ferric complexes of petrobactin and its sulfonated derivatives, highlighting differences in their complexation kinetics. Ultraperformance size-exclusion chromatography coupled with elemental and molecular MS detection, supported by quantitative NMR, enabled accurate determination of the siderophore concentration and purity in *M. nauticus* fractions. The developed method using isotopically enriched ^57^Fe and citrate as the iron source proved effective for reproducing natural speciation under environmentally relevant, slightly alkaline conditions. Petrobactin showed the least isotopic exchange with heavier iron isotopes, while disulfonated petrobactin displayed a slower reactivity. The combined applications of SEC-ICP-MS, SEC-ESI-MS, and RP-ESI-MS provided complementary insights into complex formation, speciation, and quantification.

## Supplementary Material



## Notes

Extended materials and methods; extended results and discussion; reversed-phase ESI-MS XIC chromatograms of isotopically enriched petrobactin and sulfonated petrobactin standards separated in acidic conditions (pH ≈ 2.7) (Figure S1); size-exclusion ESI-MS XIC chromatograms of petrobactin (A), sulfonated petrobactin (B), and disulfonated petrobactin (C), after spiking with ^57^Fe- and ^58^Fe-enriched standard (Figure S2); the effect of RF lens on the ionization of petrobactin (left panel) and its iron complex (right panel); *m*/*z* of 719 and 360 correspond to [M + H]^+^ and [M + 2H]^2+^ of apo petrobactin, respectively (summaric area was shown in full blue square); *m*/*z* of 772–774 and 386–387 correspond to [M + H]^+^ and [M+2H]^2+^ of ^56^Fe–^57^Fe–^58^Fe, respectively (summaric area was shown in full blue circle) (Figure S3); the effect of postcolumn ACN flow rate, RF lens, and vaporizer temperature onto ionization efficiency of apo and ferric forms of petrobactin (Figure S4); mass spectra of disulfonated petrobactin after the complexation with ^57^Fe/^58^Fe: *m*/*z* 879.275 of [M + H]^+^ and *m*/*z* 901.257 for [M + Na]^+^, after RP-ESI-MS, in acidic conditions, pH ≈ 2.7 (left panel) and its isotopically enriched complexes *m*/*z* 933.1867/934.185 for [M + H]^+^ for ^57^Fe and ^58^Fe, respectively, after separation by SEC-ESI-MS in pH 8 (Figure S5); size-exclusion ICP-MS chromatograms of ^57^Fe–citrates and ^57^Fe–malates (50 ppb) prepared in different pH of ammonium acetate (pH 5–6 and 7–8) incubated with petrobactin–sulfonated petrobactin mixture (50 μL) and ammonium acetate of pH 8.0, 8.7, and 9.5 (Figure S6); overview of variation of FeCit (orange points) and FePB (blue points) signals’ area as a function of incubation time depends on iron source concentration. Data for the system of 100 μL of petrobactin–sulfonated petrobactin mixture with 25, 50, and 100 ppb of ^57^FeCit, respectively, from left to right (Figure S7); size-exclusion ICP-MS chromatograms of the petrobactin–sulfonated petrobactin mixture (50 μL) with ^57^Fe citrates (50 ppb) over incubation time (left panel); relation of the surface area of individual signals, FeCit (A), FePBS (B), FePB (C), to the incubation time (right panel) (Figure S8); size-exclusion ICP-MS chromatograms of the petrobactin–sulfonated petrobactin mixture (100 μL) with ^57^Fe citrates (25 ppb) over incubation time (left panel); relation of the surface area of individual signals, FeCit (A), FePBS (B), FePB (C), to the incubation time (right panel) (Figure S9); size-exclusion ICP-MS chromatograms of the petrobactin–sulfonated petrobactin mixture (100 μL) with ^57^Fe citrates (100 ppb) over incubation time (left panel); relation of the surface area of individual signals, FeCit (A), FePBS (B), FePB (C), to the incubation time (right panel) (Figure S10); one H NMR spectra of petrobactin and petrobactin sulfonate (Fraction 1), MeOD at 25 °C (Figure S11); 1 H NMR spectra of petrobactin sulfonate (Fraction 2), MeOD at 25 °C (Figure S11) (PDF)

## ∥

University of Bordeaux, CNRS, Bordeaux INP, CBMN, UMR 5248, F-33600 Pessac, France
