# Supplementary material for: Reactivity of Petrobactin and Its Sulfonated Derivatives with Iron and Their Determination by Isotopic Saturation Fast Size-Exclusion Chromatography–Inductively Coupled Plasma Mass Spectrometry (ICP-MS)
Source: ACS Omega. 2025 Aug 27;10(35):40521–33. doi: 10.1021/acsomega.5c06088 (PMC12423834; doi:10.1021/acsomega.5c06088)
Supplement: Supplementary file 1 [file ao5c06088_si_001.pdf]

Supplementary information/materials

Reactivity of petrobactin and its sulfonated derivatives with iron and their determination by isotopic saturation fast size-exclusion chromatography – inductively coupled plasma mass spectrometry (ICP-MS)

Katarzyna Kińska [a,b*], Isaura Caceres[b,c], Abdel Khoukh[b], Sophie Nolivos[b], Régis Grimaud[b], Laurent Ouerdane[b], Joanna Szpunar[b], Ryszard Łobinski[b,d]

[a] University of Warsaw, Faculty of Chemistry, Pasteura 1, 02-093 Warsaw
[b] Institute of Analytical Sciences and Physico-Chemistry for Environment and Materials (IPREM-UMR5254) UPPA / CNRS, Hélioparc, 2, av. Pr. Angot, Pau, 64053, France
[c] Present adress : University of Bordeaux, CNRS, Bordeaux INP, CBMN, UMR 5248, F-33600 Pessac, France
[d] Chair of Analytical Chemistry, Faculty of Chemistry, Warsaw Institute of Technology, ul. Noakowskiego 3, 00-664 Warszawa

MATERIALS AND METHODS

Petrobactin purification.

M. nauticus SP17 was cultivated for five days at 30°C with shaking at 150 rpm in Hickford iron-free medium The medium was composed of 0.37 mol L-1 sodium chloride, 0.002 mol L-1 magnesium sulfate heptahydrate; 0.02 mol L-1 ammonium chloride; 0.02 mol L^{-1} dipotassium phosphate, 0.001 mol L-1 calcium chloride dihydrate, 0.04 mol L-1 sodium succinate adjusted to pH 7. To minimize iron contamination, the culture medium was treated with 20 g L^{-1} of ion exchange resin Chelex 100 (Bio-Rad), and all culture containers were washed with a 1 N HCl. After incubation, the culture was centrifuged at 7000 rpm for 30 min at 10°C. The supernatant was acidified to pH 5 with a 1N HCl solution. Amberlite XAD-2 resin was added to the supernatant at 50 g L^{-1}, and the mixture was shaken in the dark at 50 rpm for 12 h at room temperature . The resin was subsequently loaded into a glass column (1.5 x 20 cm), washed with three bed volumes of distillated water, petrobactin was eluted using two bed volumes of HPLC-grade methanol. The methanol fraction was tested for the presence of petrobactin using the CAS assay (Schwyn and Neilands, 1987). The methanol fraction was evaporated at 40°C under 200 mBar and the resulting dry extract was dissolved in 5 mL in double distillated water and stored at -20°C . Petrobactin was further purified by reverse-phase chromatography usig a Jupiter C4 column (5 µm ST 10/250, wide pore 300 A, Phenomenex). Elution was performed with a linear gradient from 10% to 60% acetonitrile in 0.1% trifluoroacetic acid in water. The CAS-positive fractions were evaporated to remove solvent residues, lyophilized, and dissolved in distillated water.

NMR determinations - purity and concentration. A robust method was developed for the absolute quantification of petrobactin and its sulfonated derivatives using qNMR based on the ERETIC2 (Electronic Reference To access In vivo Concentrations) approach. As described by Wider and Dreier **(2006)**, the ERETIC2 method—based on the PULCON (Pulse Length–based Concentration determination) principle—allows external referencing, thereby simplifying sample preparation. Technique developed by Bruker, enables external referencing without the use of internal standards. Ibuprofen was selected as the external reference compound. All measurements were carried out in deuterated methanol (CD_3OD), using identical NMR acquisition parameters for both reference and sample solutions to ensure consistent quantification. NMR experiments were performed on a Bruker Avance Neo 400 MHz spectrometer equipped with a 5 mm BBO SmartProbe and z-gradient pulse field. Standard 5 mm NMR tubes were used, and all measurements were conducted at 298 K. Data acquisition and processing were performed using TopSpin 4.1 (Bruker). NMR Acquisition Parameters: Pulse sequence: zg30, Relaxation delay (D1): 20 s, Spectral width (SW): 20 ppm, Acquisition time (AQ): 4.19 s, Flip angle (P1): 90° (~8.0 µs), Dummy scans (DS): 2, Number of scans (NS): 64, Spinning: OFF, Receiver gain (RG): Set automatically by TopSpin. To ensure optimal quantitative conditions, the 90° pulse length was calibrated, and the sample was automatically tuned and matched. The longitudinal relaxation times (T_1) of the aromatic protons in both ibuprofen (1.5 – 3.7) and petrobactin (2.7 – 3.0) were determined using the inversion-recovery pulse sequence in TopSpin. Accurate T_1 determination is critical for

setting an appropriate relaxation delay (D1), typically five times the longest T_1 value, to ensure complete magnetization recovery between scans. Based on these measurements, a relaxation delay (D1) of 20 s was chosen. The acquired spectra were processed using a 0.3 Hz exponential line broadening and zero-filling. Manual phase and baseline corrections were applied. Peak integration was carried out manually, with no slope or bias correction. Quantification was performed using the PULCON method in TopSpin 4.1 software, based on the integral values, reference concentration, and the number of protons corresponding to each signal.

3.1. Formation of the Fe-PB complex: preliminary experiments

3.1.1. Infusion and reversed-phase HPLC – ESI-MS

As described in the main manuscript, reverse-phased chromatographic separation with mobile phase of pH 8 allowed the detection of both the apo- and complexed forms of petrobactin and its sulfonated derivative (Fig. 3). However, at this pH, both siderophores and their complexes were very poorly retained on the RP column. A similar effect was observed when attempting to retain the complex on the SPE column, which uses a hydrophilic-lipophilic balanced reversed phase sorbent (HLB). When the FePB and FePBS complexes prepared at pH 8 were introduced into the SPE column, significantly more of the compounds was found in the solution passing through the column (90% and 85%, respectively), and only a fraction after elution with the MeOH solution (10% and 15%). A test carried out for apo forms at pH 2 and pH 8 showed that in alkaline media the sulfonated form passes through the column to a lesser extent than petrobactin (65% and 82%, respectively). At acidic pH, both compounds sorb quantitatively and are eluted by more than 99% in the next step (MeOH / 0.3% FA). The use of SPE therefore requires the sample to be acidified before it is introduced onto the column. Hence, purification/pre-concentration of petrobactin and its derivatives on SPE, proposed elsewhere for the desalting (**Boiteau et al., 2013**), is limited to apo forms, as the acidification step, necessary for the quantitative binding of compounds prevents observation of Fe-bound forms. Hence, it was necessary to find an alternative chromatographic method, allowing both the separation of individual siderophores and their separation from the matrix (matrix simplification).

3.1.2. Fast size-exclusion chromatography ESI-MS

In the first attempt, after mixing PB with the $^{57}Fe/^{58}Fe$ isotope-enriched citrate complex, the sample was injected onto the SEC column and the influence of RF lens settings on ionization was checked (Fig. S3). Raising RF up to 55-60 resulted in an increase in signal for m/z 719.4 corresponding to $[M+H]^+$ of the apo form of PB, as well as for *m/z* corresponding to double charged ion $[M+2H]^{2+}$ of petrobactin 360.2, and m/z of $[M+H]^+$ for complexed petrobactin - 772.3, 773.3, 774.3, with a simultaneous decrease of doubly charged pseudomolecular ions derived from complexes with iron: m/z 386.6, 387.1, 387.6. At RF 100, only a slight decrease was observed for the m/z of $[M+H]^+$ ions, while decrease for $[M+2H]^{2+}$ was significant.

In further optimisation, the influence of several parameters on ionization was evaluated (Fig. S4). Emphasis was placed on the impact of post-column acetonitrile flow on siderophore ionization. In the initial conditions, the acetonitrile flow matched that of ammonium acetate eluent (300 µL min^{-1}) and was subsequently reduced to 100 µL min^{-1}. As the flow decreased, a slight decrease in the signal for apo PB form was observed, with a simultaneous increase in the signal for ferric PB. The impact of RF was then checked at a reduced flow rate. Initially, the increase of RF (from 25 to 30) resulted in an increase of both forms of siderophores ($[M+H]^+$ + $[M+2H]^{2+}$). However, a further increase (up to 45) led to a further increase in the signal of the apo form, while the signal of the ferric form decreased. An increase in RF resulted in a decrease in the $[M+2H]^{2+}$ signal, while $[M+H]^+$ continued to show growth. Consequently, the RF was set at 30, as it minimized the dissociation of the complex. Finally, the effect of vaporizer and ion transfer tube temperatures were checked for the optimum RF lens settings. At constant ion transfer tube (ITT) temperature of 300°C, a change of vaporizer temperature (VT) from 300 to 350°C resulted in slight increase in all signals, with maximum at 325°C. For that conditions ITT temperature was changed from

300°C to 325°C, but negative impact was noticed (↗ apo and ↘ferric), so ITTT was kept at 300°C. In addition, to ensure optimum ionization conditions, the possibility of using post-column slightly alkalinized ACN (NH_3 addition) or methanol solution was tested. The results show that both petrobactin and its sulfonated derivative are better ionised in the presence of methanol.

3.2. Effect of the iron source on the acquisition of iron by petrobactin and its derivatives

3.2.1 Choice of the iron source

The reason for ^{57}FeEDTA stability over other forms, resulting in a less willing formation of complexes with PB, is likely due to formation of the mixed Fe(III) complexes with EDTA and OH- (Fe(OH)EDTA and $Fe(OH)_2EDTA$) at higher pH **(Hudson et al., 1992; Sunda and Huntsman, 2003)**. At pH 8, more than 99% of Fe(III) is bound to EDTA (FeL and Fe(OH)L), and both forms have relatively high stability constants (25.22 and 17.57, respectively). Although mixed EDTA-hydroxy chelates have faster dissociation kinetics, leading to the higher dissociation constants (compare to FeEDTA), those are still lower than dissociation constants of the other chelates. The high stability of EDTA and its mixed hydroxy chelates was used as an advantage at a later stage of the research leading to siderophores quantification.

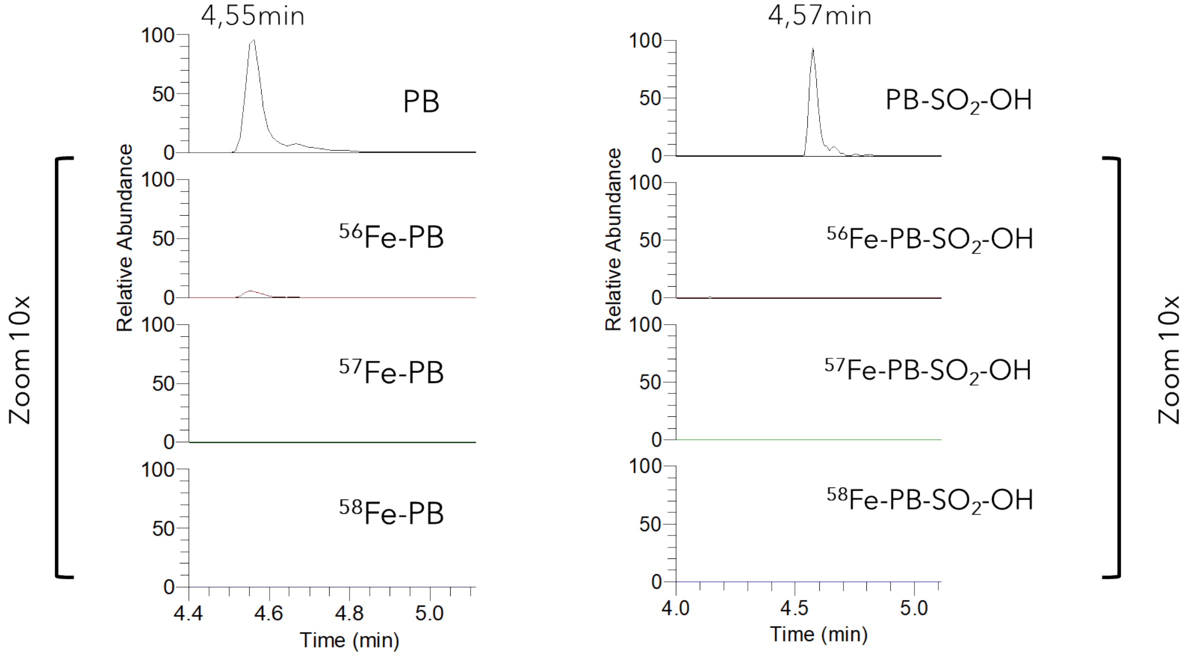

Figure S1. Reversed-phase ESI-MS XIC chromatograms of isotopically enriched petrobactin and sulfonated petrobactin standards separated in acidic conditions (pH ≈ 2.7).

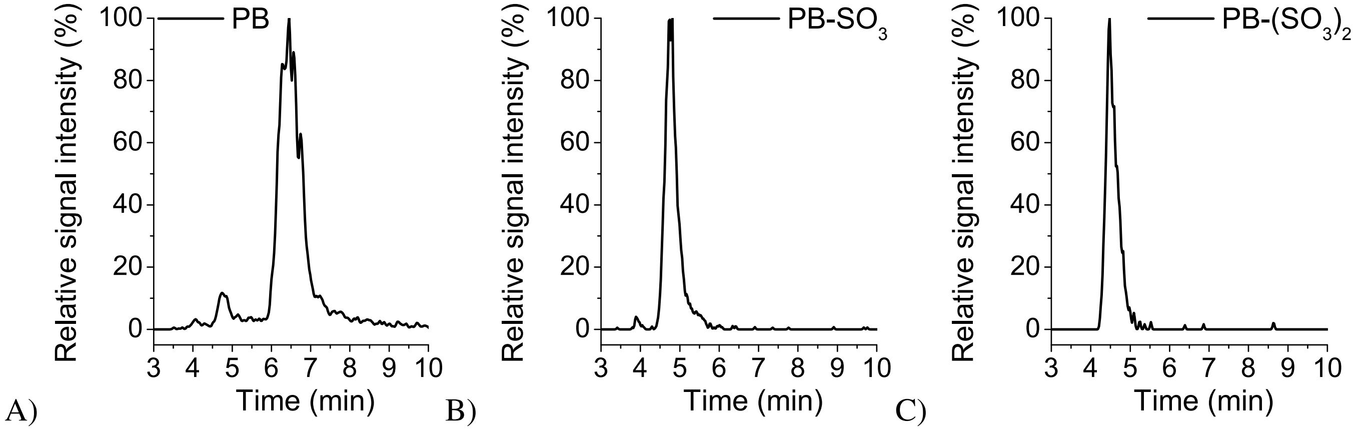

Figure S2. Size exclusion ESI-MS XIC chromatograms of petrobactin (A), sulfonated petrobactin (B) and di-sulfonated petrobactin (C), after spiking with ^{57}Fe and ^{58}Fe enriched standard.

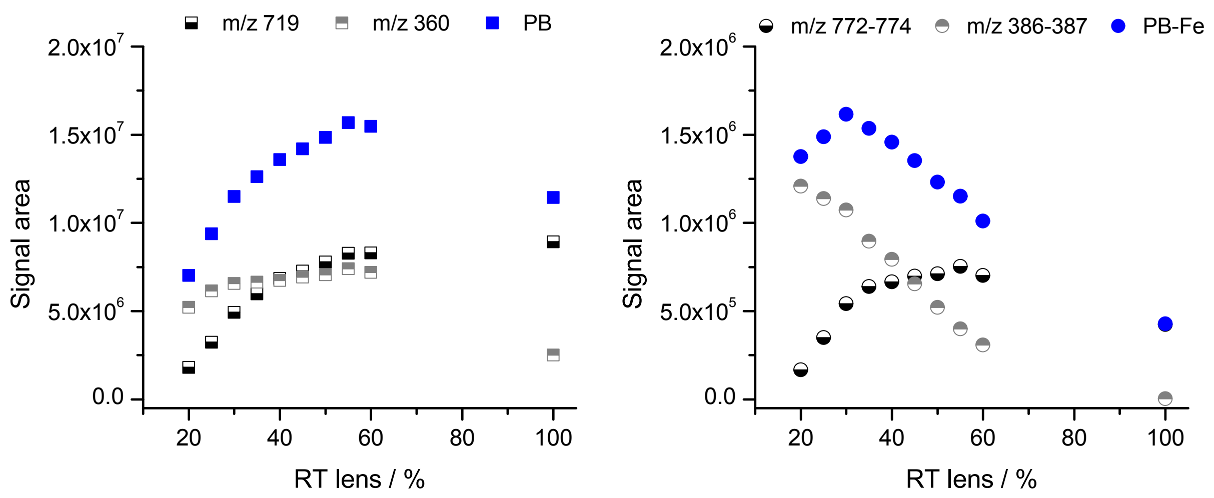

Figure S3. The effect of RF lens on the ionisation of petrobactin (left panel) and its iron complex (right panel); m/z of 719 and 360 corresponds to $[M+H]^+$ and $[M+2H]^{2+}$ of apo petrobactin, respectively (summaric area was shown in full blue square); m/z of 772-774 and 386-387 corresponds to $[M+H]^+$ and $[M+2H]^{2+}$ of ^{56}Fe-^{57}Fe-^{58}Fe, respectively (summaric area was shown in full blue circle).

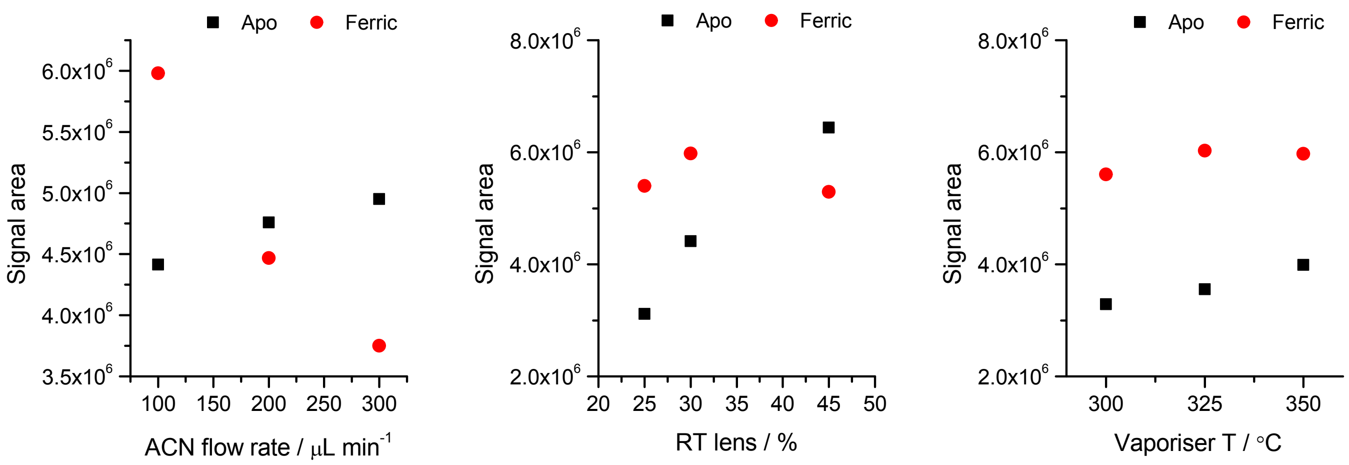

Figure S4. The effect of post-column ACN flow rate, RF lens and vaporiser temperature onto ionization efficiency of apo and ferric form of petrobactin.

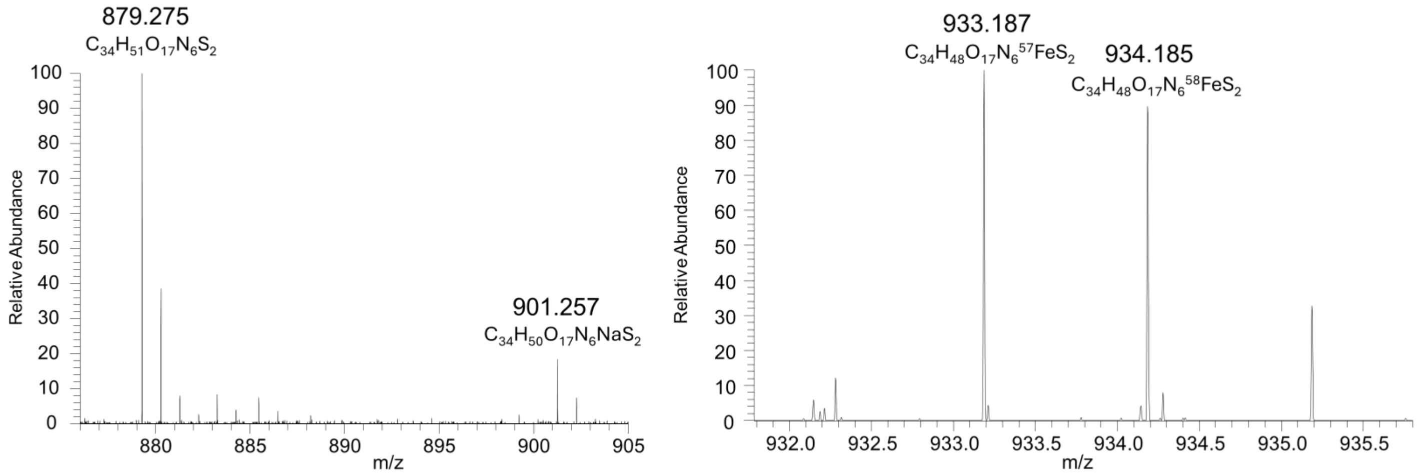

Figure S5. Mass spectra of disulfonated petrobactin after the complexation with $^{57}Fe/^{58}Fe$: m/z 879.275 of $[M+H]^+$ and m/z 901.257 for $[M+Na]^+$, after RP-ESI-MS, in acidic conditions, pH ≈ 2.7 (left panel) and its isotopically enriched complexes m/z 933.1867/934.185 for $[M+H]^+$ for ^{57}Fe and ^{58}Fe respectively, after separation by SEC-ESI-MS in pH 8.

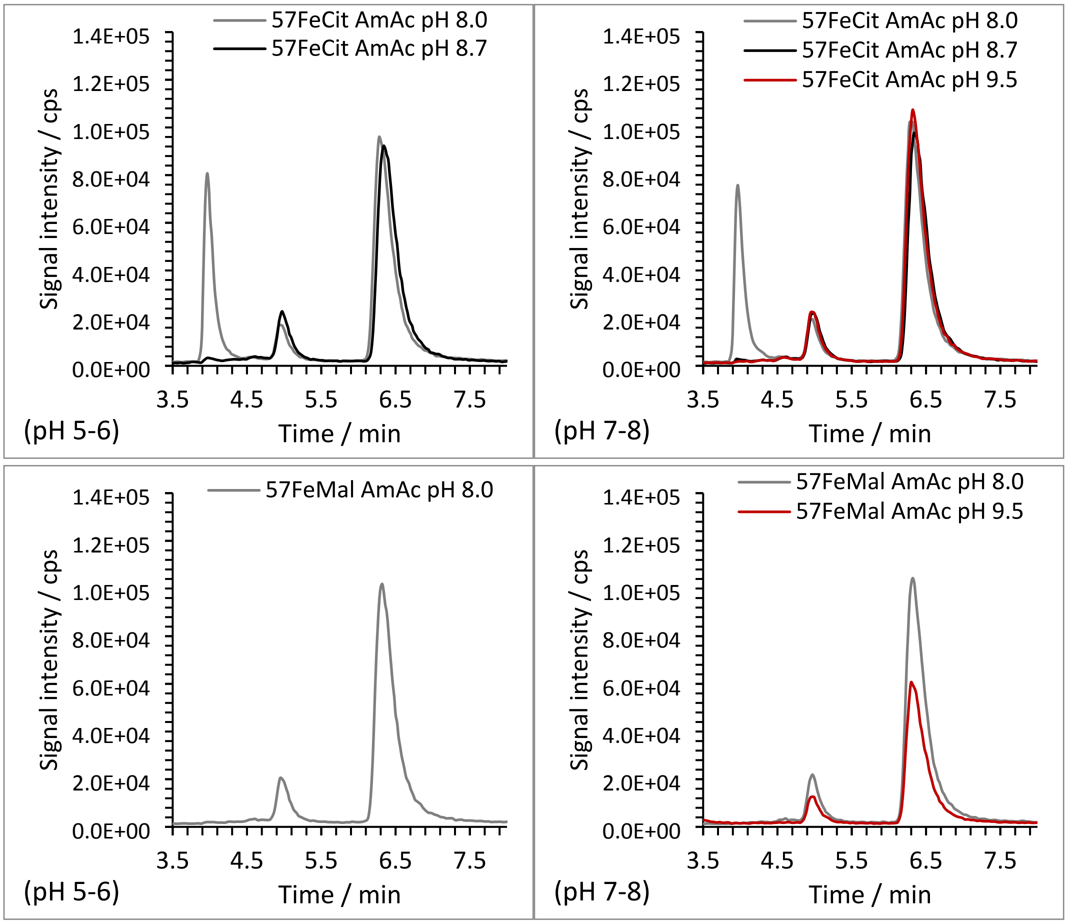

Figure S6. Size exclusion ICP-MS chromatograms of ^{57}Fe – citrates and ^{57}Fe – malates (50ppb) prepared in different pH of ammonium acetate (pH 5-6 and 7-8) incubated with petrobactin-sulfonated petrobactin mixture (50µL) and ammonium acetate of pH 8.0, 8.7 and 9.5.

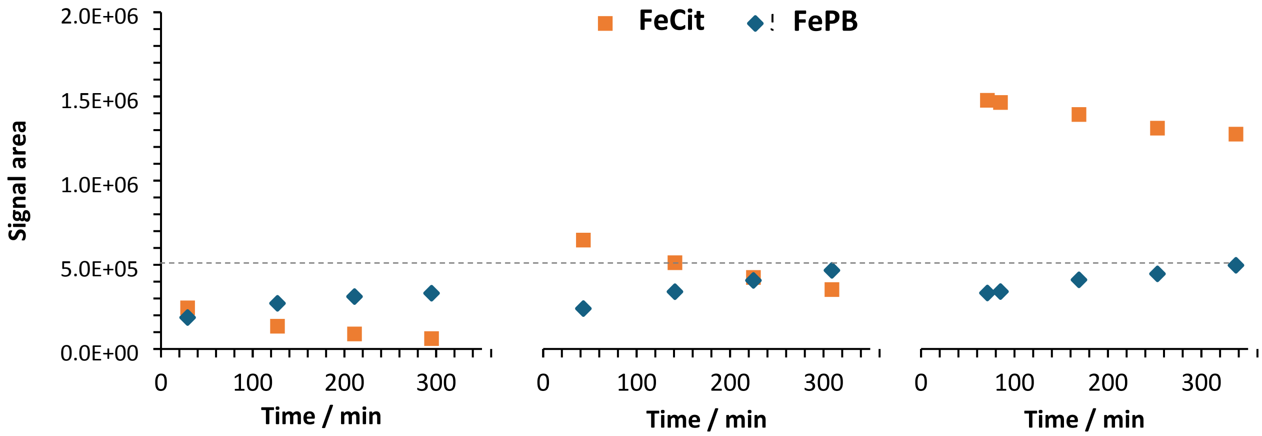

Figure S7. Overview of variation of FeCit (orange points) and FePB (blue points) signals' area as a function of incubation time depends on iron source concentration. Data for the system of 100µL petrobactin-sulfonated petrobactin mixture with 25ppb, 50ppb and 100ppb of ^{57}FeCit, respectively from left to right.

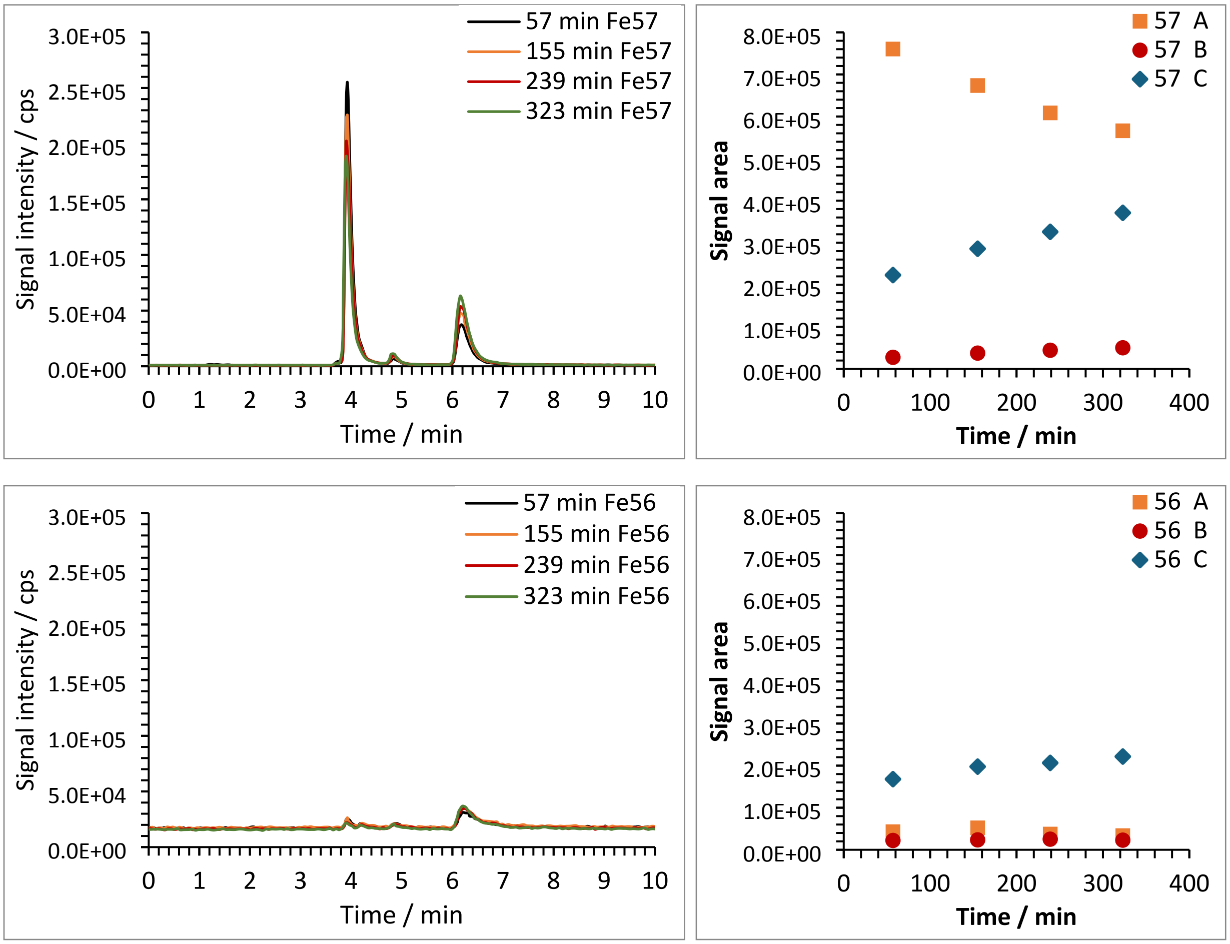

Figure S8. Size exclusion ICP-MS chromatograms of the petrobactin-sulfonated petrobactin mixture (50µL) with ^{57}Fe citrates (50ppb) over incubation time (left panel); relation of the surface area of individual signals, FeCit (A), FePBS (B), FePB (C), to the incubation time (right panel).

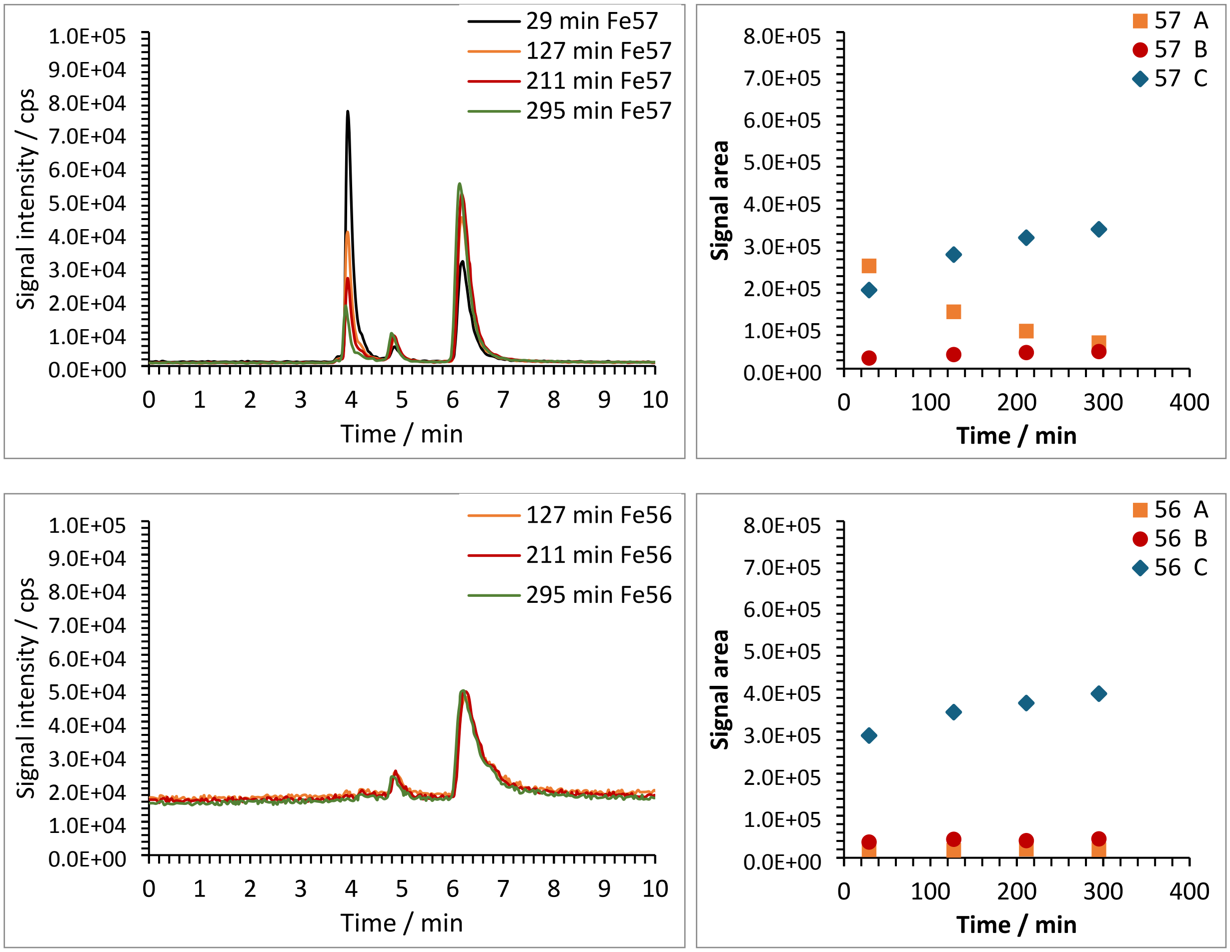

Figure S9. Size exclusion ICP-MS chromatograms of the petrobactin-sulfonated petrobactin mixture (100µL) with ^{57}Fe citrates (25ppb) over incubation time (left panel); relation of the surface area of individual signals, FeCit (A), FePBS (B), FePB (C), to the incubation time (right panel).

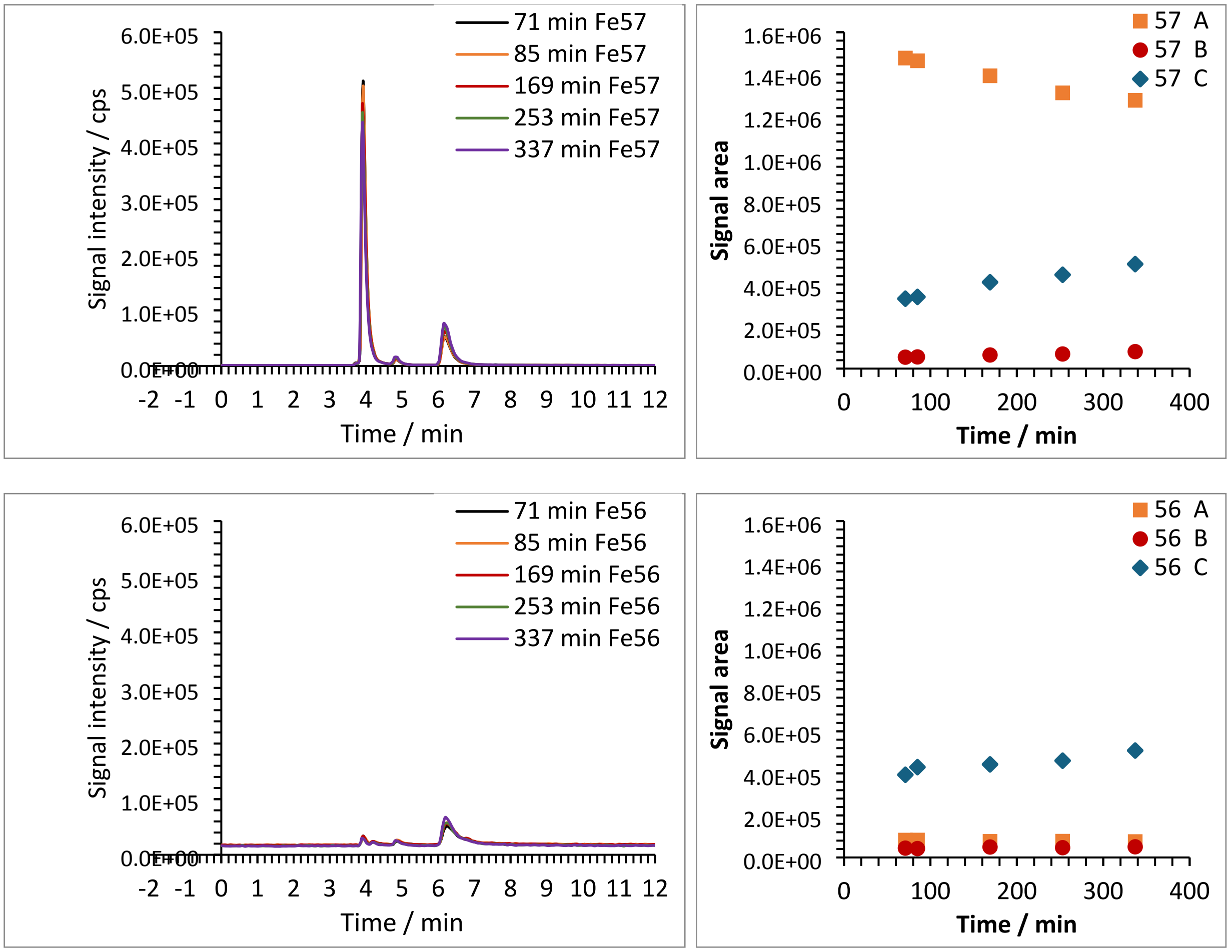

Figure S10. Size exclusion ICP-MS chromatograms of the petrobactin-sulfonated petrobactin mixture (100µL) with ^{57}Fe citrates (100ppb) over incubation time (left panel); relation of the surface area of individual signals, FeCit (A), FePBS (B), FePB (C), to the incubation time (right panel).

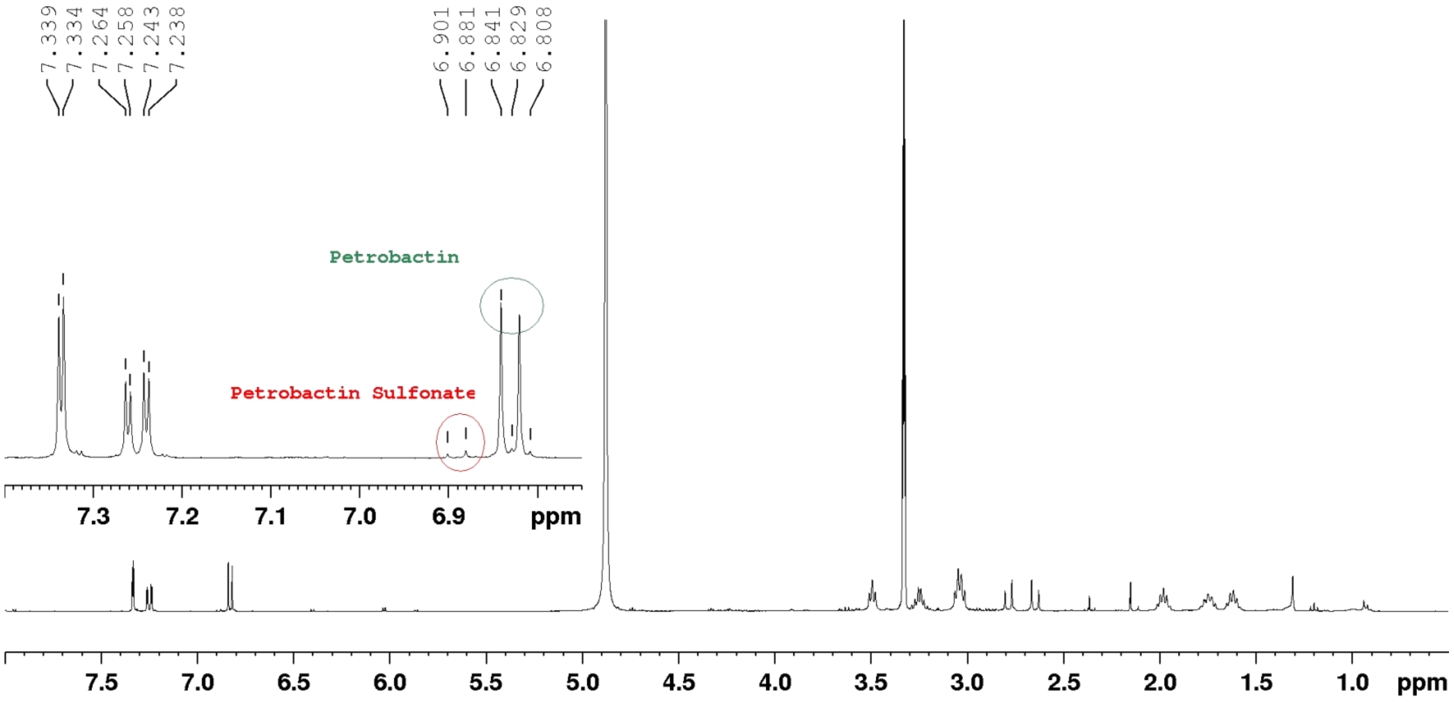

Figure S11. [1] H NMR spectra of petrobactin and petrobactin sulfonate (Fraction 1), MeOD at 25˚C

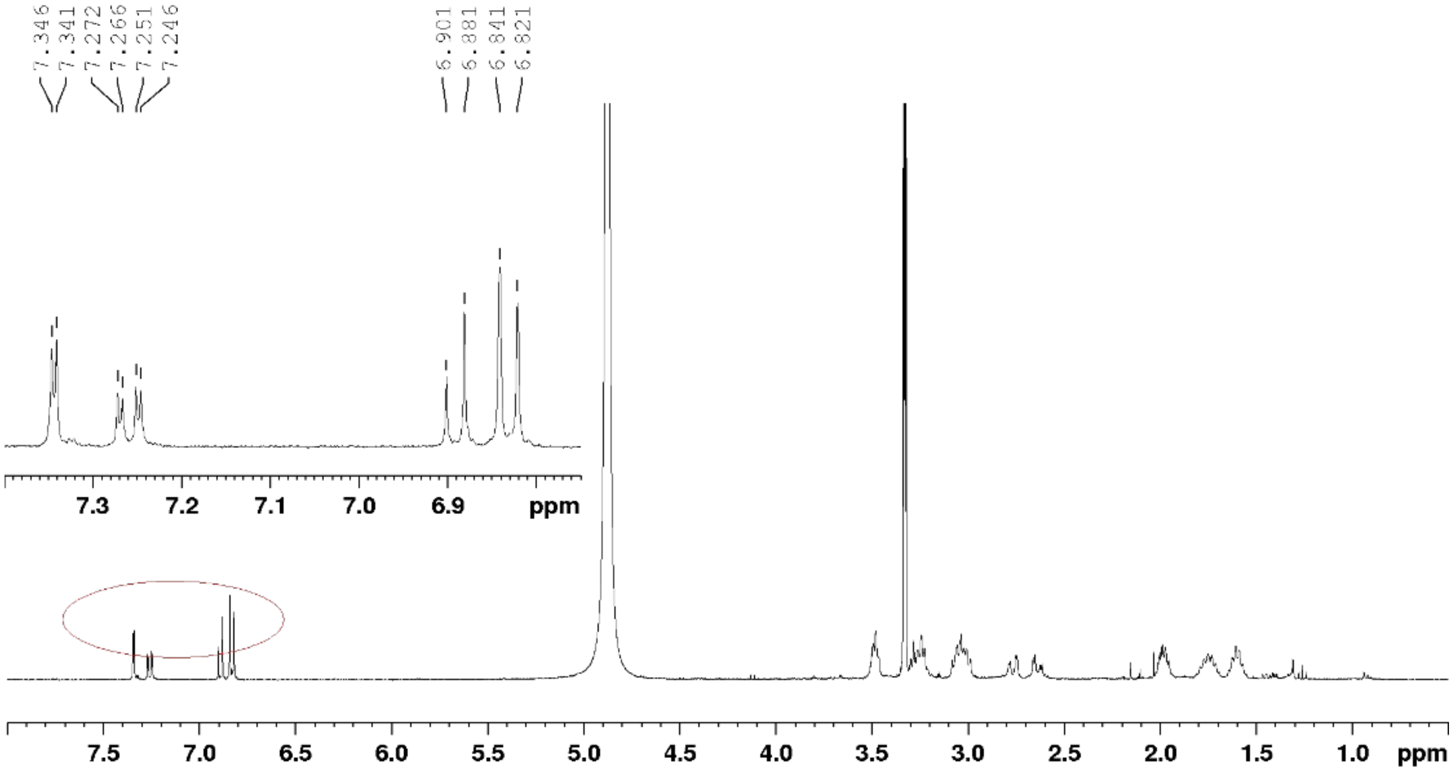

Figure S12. [1] H NMR spectra of petrobactin sulfonate (Fraction 2), MeOD at 25˚C
